# Supervised exercise following bariatric surgery in morbid obese adults: CERT-based exercise study protocol of the EFIBAR randomised controlled trial

**DOI:** 10.1186/s12893-019-0566-9

**Published:** 2019-09-05

**Authors:** Emilio Villa-González, Yaira Barranco-Ruiz, Manuel A. Rodríguez-Pérez, Alejandro Carretero-Ruiz, José María García-Martínez, Alba Hernández-Martínez, María José Torrente-Sánchez, Manuel Ferrer-Márquez, Alberto Soriano-Maldonado, Enrique G. Artero, On behalf of the EFIBAR Study Group

**Affiliations:** 1 Department of Physical and Sports Education, PROFITH “PROmoting FITness and Health through Physical Activity” Research Group, Sport and Health University Research Institute (iMUDS), Faculty of Education and Sport Sciences, University of Granada, 52005 Melilla, Spain; 2 Department of Education, Faculty of Education Sciences, University of Almería, Almería, Spain; 3 SPORT Research Group (CTS-1024), CERNEP Research Center, University of Almería, Almería, Spain; 4 Hospital Mediterráneo, Almería, Spain; 5 Torrecárdenas University Hospital, Almería, Spain

**Keywords:** Obese, Exercise programmes, Bariatric surgery, Adult and weight regain

## Abstract

**Background:**

There is increasing evidence of weight regain in patients after bariatric surgery (BS), generally occurring from 12 to 24 months postoperatively. Postoperative exercise has been suggested to ad long-term weight maintenance and to improve physical function in BS patients. However, there are a limited number of intervention studies investigating the possible benefits of exercise in this population. The aim of the current report is to provide a comprehensive CERT (Consensus on Exercise Reporting Template)-based description of the rationale and details of the exercise programme implemented in the EFIBAR Study (*Ejercicio FÍsico tras cirugía BARiátrica*), a randomised controlled trial investigating the effects of a 16-week supervised concurrent (aerobic and strength) exercise intervention program on weight loss (primary outcome), body composition, cardiometabolic risk, physical fitness, physical activity and quality of life (secondary outcomes) in patients with severe/morbid obesity following bariatric surgery.

**Methods:**

A total of 80 BS patients [60–80% expected women, aged 18 to 60 years, body mass index (BMI) ≥ 40 kg/m^2^ or ≥ 35 kg/m^2^ with comorbid conditions)] will be enrolled in the EFIBAR Randomized Control Trial (RCT). Participants allocated in the exercise group (*n* = 40) will undertake a 16-week supervised concurrent (strength and aerobic) exercise programme (three sessions/week, 60 min/session), starting 7 to 14 days after surgery. The rationale of the exercise programme will be described following the CERT criteria detailing the 16 key items. The study has been reviewed and approved by the Ethics Committee of the Torrecárdenas University Hospital (Almería, Spain) (ref. N° 76/2016).

**Discussion:**

The present study details the exercise programme of the EFIBAR RCT, which may serve: 1) exercise professionals who would like to implement an evidence-based exercise programme for BS patients, and 2) as an example of the application of the CERT criteria.

**Trial registration:**

The trial was prospectively registered at Clinicaltrials.gov NCT03497546 on April 13, 2018.

**Electronic supplementary material:**

The online version of this article (10.1186/s12893-019-0566-9) contains supplementary material, which is available to authorized users.

## Background

Bariatric surgery (BS) is an effective weight loss intervention for morbidly obese patients, being successful in the treatment of obesity (stage I and II) and different associated diseases [1]. Nevertheless, there is increasing evidence of weight regain in patients after BS, [2, 3] usually occurring between 12 and 24 months postoperatively [4, 5]. Weight regain increases the risk of physical function decline, which negatively affects the functionality of an individual to carry out tasks of daily life [5]. Weight regain also increases the likelihood of obesity-related comorbidity relapse [6], which makes it necessary to implement strategies to improve the lifestyle before [7] and after BS [8].

Postoperative lifestyle interventions combining diet, exercise and behaviour modification have proven successful in aiding long-term weight maintenance and improving physical function in BS patients [9]. However, there are a limited number of intervention studies investigating the possible multidimensional benefits of exercise in this population. For patients who have undergone BS and experience suboptimum weight loss, exercise could be an important adjunct therapy [10, 11]. A previous review found a positive effect of exercise on anthropometric measurements, cardiovascular risk factors and physical fitness in BS patients [12], although results were not consistent, with a wide range of exercise programmes and perioperative timing, therefore hampering adequate practical guidance. Despite this heterogeneity, authors concluded that a beneficial exercise programme should last for a median of 12 weeks, have a mean intensity of 65% peak heart rate/VO_2max_, and be at least partially supervised [12]. A recent metha-analysis concluded that exercise training programs performed after BS were effective to optimize weight loss and fat mass loss and to improve physical fitness, although no additional effect on lean body mass loss was found [13].

In particular, strength training increases muscular strength and attenuates muscle atrophy in obese adults adhering to caloric restriction for weight loss [14, 15], whereas aerobic training provides several metabolic improvements, such as attenuating the appearance of arterial stiffness [16]. Unfortunately, it is difficult to assess the appropriate type and amount of exercise in BS patients, as it is a relatively understudied population and a variety of training regimes have been described. To date, published evidence supports a potential role for exercise to elicit positive changes in body composition after BS. However, properly designed exercise-based randomised controlled trials (RCT) are needed to provide further evidence of the effectiveness of exercise as a feasible adjunct therapy to BS [17].

In general, descriptions of exercise intervention programmes in clinical research have traditionally been vague, often containing insufficient information to allow for replication. To address this, The Consensus on Exercise Reporting Template (CERT) was recently developed to standardise the reporting of exercise intervention programmes regardless of the population [18] CERT recommends that exercise programmes published in the literature should ideally report all of the components considered in the intervention, since these are essential to evaluate the effects of clinical trials, and the interpretation, translation and implementation of research findings into clinical practice. The Consensus provides guidance on a minimum set of 16 key items required to report replicable exercise programmes. While its development was stimulated by a metaepidemiological review of exercise interventions for chronic health conditions [19] it is equally applicable to describe exercise interventions for acute conditions, injury prevention or general health. Additionally, a recent report highlighted the importance of describing exercise programs following CERT recommendations, to facilitate the replicability of these programs in BS population [20].

The aim of the current report is to provide a comprehensive CERT-based description of the rationale and details of the exercise programme implemented in the EFIBAR (*Ejercicio FÍsico tras cirugía BARiátrica*) study, a 1-year RCT investigating the effects of a 16-week supervised concurrent (aerobic and strength) exercise intervention program on weight loss (primary outcome), body composition, cardiometabolic risk, physical fitness, physical activity and quality of life (secondary outcomes) in patients with severe/morbid obesity following bariatric surgery.

## Methods/design

### Study design

Briefly, a total of 80 BS patients [60–80% expected women, aged 18 to 60 years, body mass index (BMI) ≥ 40 kg/m^2^ or ≥ 35 kg/m^2^ with comorbid conditions)] will be enrolled in the EFIBAR Randomized Control Trial (RCT) (ClinicalTrials.gov ID: NCT03497546), following recruitment from a public hospital and a private clinic in Almería, southern Spain. Participants will be randomised either to a normal care Control Group (*n* = 40), involving nutritional status monitoring and diet/physical activity counselling following international guidelines [21], or an Exercise Group (*n* = 40), who will additionally undertake a 16-week supervised concurrent (strength and aerobic) exercise programme (three sessions/week, 60 min/session) starting 7 to 14 days after surgery, with the final aim of evaluating the effects on weight loss (primary outcome), body composition, cardiometabolic risk, physical fitness and quality of life (secondary outcomes). Recruitment of participants started in May 2018 and it may extend till December 2020.

Sample size calculations indicate that, assuming an alpha error of 0.05 and a power of 80%, a total of 66 patients (*n* = 33 patients per group) will be needed to detect an effect (between group difference) of at least 0.7 standard deviations [22] in the main outcome (% total weight loss, %TWL). Anticipating a potential lost to follow-up of up to 20%, a total of 80 patients will be recruited. Additionally, we will aim at maximizing the adherence and minimizing lost to follow-up, which could in fact increase the power to detect the main effect.

**Fig. 1:**
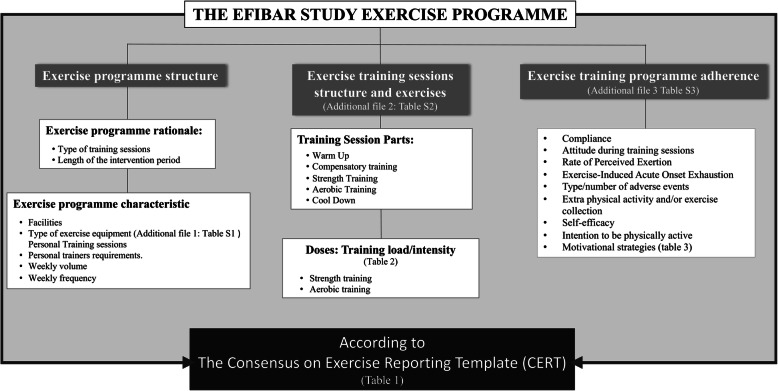
Graphical abstract of the detailed exercise programme of the EFIBAR Study

The rationale of the exercise programme implemented in EFIBAR will be described following the CERT criteria recommendations for detailing the 16 items (Table 1). The structure that was followed to describe the exercise programme is presented in the Fig. 1.

**Table 1:** CERT checklist from EFIBAR study exercise programme

	Item	Checklist Item	Identification (section)
WHAT: materials	1	Detailed description of the type of exercise equipment	Additional file 1: Table S1
WHO: provider	2	Detailed description of the qualifications, expertise and/or training	Exercise programme characteristics
HOW: delivery	3	Describe whether exercises are performed individually or in a group	Exercise programme characteristics
	4	Describe whether exercises are supervised or unsupervised; how they are delivered	Exercise programme characteristics
	5	Detailed description of how adherence to exercise is measured and reported	Programme adherence Tally sheet/daily control (Additional file 3: Table S3)
	6	Detailed description of motivation strategies	16 reinforcement WhatsApp messages / every Friday (Additional file 3) 4 reinforcement videos / end of every month (Additional file 3)
	7a	Detailed description of the decision rule(s) for determining exercise progression	Doses: Training load/intensity
	7b	Detailed description of how the exercise programme was progressed	Doses: Training load/intensity
	8	Detailed description of each exercise to enable replication	Additional file 2: Table S2
	9	Detailed description of any home programme component	Programme adherence
	10	Describe whether there are any non-exercise components	Exercise programme rationale
	11	Describe the type and number of adverse events that occur during exercise	Tally sheet/daily control (Additional file 3: Table S3)
WHERE: location	12	Describe the setting in which the exercises are performed	Exercise programme characteristics
WHEN, HOW MUCH: dosage	13	Detailed description of the exercise intervention	Weekly volume
TAILORING: what, how	14a	Describe whether the exercises are generic (one size fits all) or tailored	Sessions structure and exercises
	14b	Detailed description of how exercises are tailored to the individual	Sessions structure and exercises
	15	Describe the decision rule for determining the starting level	Sessions structure and exercises
HOW WELL: planned, actual	16a	Describe how adherence or fidelity is assessed/measured	Programme adherence Tally sheet/daily control (Additional file 3: Table S3)
	16b	Describe the extent to which the intervention was delivered as planned	Tally sheet/daily control (Additional file 3: Table S3)

### Programme structure

#### Exercise programme rationale

With the overall aim of making this exercise programme transferable to society, the exercise level in EFIBAR is based on the physical activity recommendations for adults proposed by the World Health Organization [23], together with the American College of Sports Medicine’s position stand [24] on progression models in strength and aerobic training for healthy adults. Since there is limited information regarding the ideal exercise model for morbidly obese adults, we will combine strength and aerobic training (i.e., a concurrent training protocol), as previous findings in obese adults displayed important benefits when both strength and aerobic exercise are implemented in the same session [25]. A question that often arises is whether aerobic or strength training should be performed first within any particular training session. It has been suggested that performing aerobic exercise prior to strength training could result in impaired strength training performance (e.g., strength exercises technique), and unfavourable responses to strength (decrease in force production) when strength training is conducted with moderate to high loads in the general population [26]. Moreover, in a previous study conducted in obese men, concurrent strength prior to aerobic training generated a greater effect in reducing body fat compared to aerobic prior to strength training [27]. However, further research is still needed to know which order (i.e., strength prior to aerobic or vice versa) produces greater benefits in BS patients.

The length of the intervention period is 16 weeks based on a previous large scale RCT [28], where substantial physiological adaptations occurred within the first 3 to 6 months of exercise. We also considered the increased logistical and participation burdens, which indeed might lead to poorer adherence, as well as the cost of running a highly controlled laboratory-based study for a longer period. In addition to the exercise programme, all participants (both control and exercise groups) will undergo routine visits to the clinic during medical discharge and also for follow-up visits (months 1, 3, 6, 9 and 12). In these examinations, lifestyle-specific instructions (Item 10), including dietary and physical activity recommendations, will be provided as indicated in the perioperative clinical practice guidelines by the American Association of Clinical Endocrinologists, The Obesity Society, and American Society for Metabolic & Bariatric Surgery [29].

#### Exercise programme characteristics

The exercise programme will be carried out in a fitness room at the University of Almería (400 m^2^), which is equipped with aerobic and strength training areas. The mean temperature registered in Almería from www.aemet.es averaged 19° during 2018. The training programme will be free for participants (Item 12). The detailed description of the exercise equipment (Item 1) used in the training programme is shown in Additional file 1: Table S1. Two Personal Trainers (PT) with BSc degrees in Sport Sciences, and at least 2 years of experience as personal trainers, will be in charge of all sessions (Item 2). The PTs will receive a written standardised programme and weekly instruction by the study group training coordinators. Finally, the exercise programme will be developed individually (i.e., personal training sessions) (Item 3) and always supervised with a ratio of one-to-one (1,1, personal trainer, participant) (Item 4), unless participants report schedule unavailability; if this is the case, some sessions will be permitted to include two patients per PT.

#### Weekly volume

The prescription of the exercise programme will try to meet the recommendations proposed by current guidelines for aerobic and strength training in healthy adult populations [29]. The total exercise training volume will be 180 min/week. The doses will be composed of 48 sessions (1 h/session) over 16 weeks (3 times/week). A full description of the training periodization (Item 13) is shown in Table 2.

It is estimated that 150 min/week of moderate intensity, i.e., ≈3–5.9 metabolic equivalent (MET, 1 MET = 3.5 ml O_2_/kg/min) aerobic physical activity is equivalent to 1000 kcal/week; this is associated with lower rates of cardiovascular disease and premature mortality in healthy adults [29]. Moreover, an energy expenditure of 1000 kcal/week can also be achieved with ≈75 min/week of vigorous intensity (≥6 METs) aerobic exercise. Thus, the proposed weekly volume of 180 min/week of concurrent training is likely to elicit ≈1200 kcal/week. PTs will continuously monitor heart rate using a *Polar* Electro V-800 (Kempele, Finland) in all sessions of the intervention programme.

#### Weekly frequency

Although physical activity recommendations suggest undertaking physical activity most or preferably all days of the week, there was concern that exercising more than 3 days per week would be an excessive burden and might have undesirable effects on participants’ adherence and motivation to complete the exercise intervention programme. Studies on exercise frequency show little differences for 3 or more days per week provided the weekly dose of exercise is attained [29]. Participants in the EFIBAR study will perform the exercise programme 3 days per week (preferably on Mondays, Wednesdays and Fridays). If the participant misses one training session, the participant will be rescheduled for a different day of the week to recover the session, provided the necessary between-session resting time is achieved (a minimum of 24 h). A previous study of morbidly obese women found that three sessions per week improved cardiac parameters, such as variability and heart rate kinetic [30]. At the end of each training week, the PTs will remind participants of their appointments for the following week’s training sessions to increase attendance.

#### Sessions structure and exercises

The main structure of the physical exercise sessions will be: 1) warm up; 2) compensatory training; 3) strength training; 4) aerobic training; and 5) cool down. A detailed description of the exercises used in the EFIBAR RCT (Item 8) is shown in Additional file 2: Table S2. Briefly, the warm up comprises 5 min of low intensity aerobic activity (treadmill) at 50 to 65% of heart rate reserve (HRR). Compensatory training includes core stability and stabiliser muscle exercises prior to strength training. Strength training will comprise a whole body exercise routine involving major upper and lower body muscle groups progressed through three phases after a familiarisation phase. Then, aerobic training will be conducted on a treadmill. Finally, participants will perform a cool down including static and dynamic stretching exercises.

In compensatory training, from week 3 until the end of the programme, participants will perform nine core stability and stabiliser muscle exercises, in order to minimise risk of injury and, hopefully, increase adherence (page 30 to 59 of Additional file 2: Table S2). All exercises will be modified such that they can be performed at three levels of difficulty: basic (Level A), intermediate (Level B) and advanced (Level C). All participants will begin at the basic level (Item 15). These exercises will be carefully progressed in level of difficulty (Item 7a and 7b). In general, Level A will be carried out from week 3, Level B from week 5, and Level C from week 11. A certain degree of individualization will also be permitted, as each participant will progress through the different exercise levels (i.e., A, B or C) according to their individual adaptation; when the participant successfully complete all sets/repetitions of the prescribed exercises in a level (see Additional files 2 and 3) over more than three consecutive sessions (i.e., over 1 week), they will be able to progress to the next level. Our PTs will modify all exercises based on the participant’s response to the exercise (Item 14a and 14b)*.* The progression and order has been carefully structured, as presented in Additional file 2: Table S2. The proposed exercises allow for good activation of the central area of the body (e.g., abdominal area) with low pressure in the spinal structures (e.g., bird dog, side plank or modified crunch), as recommended in previous studies [31].

For strength training*,* the exercises will progress in intensity based on the participant’s response to the exercise during the four main phases (Item 7a and 7b): familiarisation (weeks 1 to 4), phase 1 (weeks 5 to 8), phase 2 (weeks 9 to 10) and phase 3 (weeks 11 to 16). Participants will go through a familiarisation period during the first 4 weeks of the exercise program before including external loads*.* Since participants may present some movement limitations due to discomfort during the first weeks after surgery [32], we will prescribe exercises that allow participants to learn movement patterns (from week 1) and weight-bearing and strength training with elastic bands (from week 3) of the different main movements (e.g., squat, horizontal pull, vertical push); this will provide participants with the appropriate technique for the main exercises. In this phase, participants will learn seven movement pattern exercises, which constitute the basis of movement, such as diaphragmatic or abdominal breathing, dissociation and mobility of the hip or stabilization of the shoulders or the wrist. These seven exercises can be performed whenever the PT feels they are necessary over the 16 weeks in order to remind the patients of the basic movement patterns. During the first 4 weeks, participants will perform two sets of 5 to 7 repetitions of each movement pattern exercise. The resting time between series of these exercises will be adjusted to the patients’ perceptions. Moreover, they will be asked to perform the exercises at progressively increasing speeds. It will be recommended that they do not perform trunk and hip flexion exercises until week 2 or 3 to avoid discomfort in the area of the surgical intervention (see the example of the hip flexion exercises 2 and 6 of movement pattern exercises in Additional file 2: Table S2). Likewise, in order to minimise trunk flexion during the first 2 to 3 weeks, we will avoid upward facing positions by proposing foot variants (see exercises 1 and 2 of the movement patterns exercises). Given the participants’ degree of obesity, the order of the exercises was carefully designed to improve the flow of the sessions (i.e., reducing the number of transitions from lying to standing positions). Thus, the seven proposed exercises will be performed in the order detailed in Additional file 2: Table S2.

From phase 1 (week 5) onwards, all participants will perform exercises with external loads, including six main exercises performed in the following order: 1) squat; 2) seated lat pull-down; 3) bench press; 4) seated low row; 5) push press with dumbbells; and 6) deadlift. The training progression from one exercise to another will be done vertically (e.g., exercise 1 – rest – exercise 2). A detailed description of each exercise is presented in Additional file 2: Table S2, according to previous training recommendations [4] (Item 14a and 14b). Participants will be instructed to exercise through the full range of motion and to avoid the Valsalva manoeuvre. Finally, it should be noted that previous studies used similar strength training modalities in BS populations, such as stack-weight equipment, free weights, body weight or resistive bands, in order to maintain high levels of enjoyment, engagement, and most importantly, adherence [33].

The aerobic training part will be conducted on a treadmill after strength training. Previous findings recommend that obese individuals can briskly walk or even run, provided they follow conservative transitions and progression, schedule rest days and heed onset of pain symptoms [34]. For progression to running, intensity or mileage increases should be slow and consistent to prevent musculoskeletal injury. Moreover, a study by Vincent et al. indicated that patients who have undergone bariatric surgery and are now lean can also run, but special foci, such as hydration and energy replacement, must be considered. All these considerations will be taken into account in the EFIBAR RCT.

Finally, participants will perform a cool down, including 5 min of static and dynamic stretching exercises in order to promote training adherence.

#### Doses: training load/intensity

Several public health institutions clearly indicate that moderate intensity physical activity is beneficial for health in deconditioned persons [23, 35], yet additional benefits have been observed for vigorous compared to moderate intensity exercise [29]. An intensity of at least 60% HRR is sufficient to produce clinically significant physiological adaptations in sedentary individuals [23, 29, 36]. We are aware that our participants might not be immediately capable of exercising at their required volume and intensity dose; therefore, there will be a gradual progression to the assigned exercise dose. The training loads in the EFIBAR RCT will range from ≈50 to 75% of one repetition maximum (1 RM) for strength training, and from 65 to 85% HRR for aerobic training. Moreover, the Borg Rating of Perceived Exertion (RPE) scale will also be used to monitor the intensity of aerobic training [36] (intensity values from 6 to 9, where the scale is 1–10) and the OMNI-resistance scale (intensity values from 5 to 8, where the scale is 1–10) will be used for strength training [37].

##### Strength training

An intensity equivalent to 40–50% of 1 RM may be beneficial for improving muscle strength in sedentary persons beginning a strength training programme, whereas 60–70% of 1 RM is recommended for novice to intermediate exercisers to improve strength [24]. The main strength training phase (i.e., from weeks 5 to 16) will have an intensity progressing from 24 to 10 RM (≈50 to 75% of 1RM), quantified by perceived exertion (CE) as in previous studies [38, 39] (i.e., the maximum number of repetitions which participants could perform with a given load). In week 5, participants will perform one set of 12 repetitions of strength training, lifting a load with which the participant could perform a maximum of 24 repetitions, whereas participants will finish the programme (week 16) performing three sets of 6 repetitions using the load corresponding to 10 maximum repetitions. CE will be readjusted twice during the exercise programme: at the beginning of phase 1 (session 13) and during phase 3 (session 31).

As the load (i.e., % RM) is not the only variable that determines strength training intensity, we will control other variables such as movement speed during both concentric and eccentric phases, recovery time and range of motion; therefore, we assume that different loads (i.e., ≈50% RM vs. 75% RM) will constitute different training intensities [24, 40]. The cadence for the strength exercises is fixed at 1:2 (concentric: eccentric phases), performing the concentric phase with the maximum velocity possible [41]. On the other hand, it is well known that different configurations of the strength training stimulus can elicit different physiological responses (e.g., muscle damage, metabolic stress) [42]. Taking into account that it is not known which kind of strength training stimulus is best for this population, we will vary the type of stimuli across the three different strength training phases. From weeks 5 to 8, we will conduct strength training based on metabolic stress (i.e., <load and > volume), from weeks 9 to 11 it will be based on metabolic stress/mechanical tension and muscle damage, and from week 12 to the end of the intervention we will conduct a programme based on mechanical tension and muscle damage stress (i.e., >load and < volume), following previous strength training recommendations [40, 42]. For strength training with elastic bands (weeks 3 and 4), the intensity will be controlled by the Thera-Band RPE scale (range 2 to 6, easy to somewhat hard) [43]. Rest periods between sets were intentionally minimised to 30 to 60 s to achieve the proposed intensity [44].

##### Aerobic training

Aerobic training intensity will be controlled based on heart rate reserve (HRR). HRR will be calculated from the maximum real heart rate (HRmax) achieved during the maximum treadmill test, which will be performed at baseline as part of the pre-surgery evaluations. The Karvonen formula ([(maximum heart rate – resting heart rate) x % training sensitive zone] + resting heart rate) will be used to calculate the individual exercise intensity. Resting heart rate will be taken from the heart rate variability (HRV) assessment included in the pre-surgery evaluations, as this test requires 10 min of sitting rest. Five training zones will be used: Zone 1 < 55–69%; Zone 2: 70–79%; Zone 3: 80–84%; Zone 4: 85–89%; and Zone 5: > 90%. Heart rate monitors will be programmed according to the percentage of the participants individual HRRmax. In order to adjust the individual exercise intensity throughout the programme, the HRR will be estimated using the Karvonen formula in weeks 12 and 36. In addition, prior to the beginning of each exercise session, participants will wear a Polar heart rate monitor (V-800) and rest in a seated position for 5 min, after which time their heart rate will be recorded to ensure that they are not beginning with a heart rate in high training zones [44].

Participants will start with a dose of 15 min/session of aerobic training with ≤65% HRR during weeks 1 to 4 (familiarisation phase). From this point onwards, they will start a gradual increase in exercise volume and intensity. The volume will increase to 20 min/session from 65 to 70% of HRR during weeks 5 to 8 (phase 1). The volume in phase 2 will be maintained (20 min/session), but with an increase of 5% in intensity (i.e., until 75% of HRR). The first week of phase 3 will have the same volume as phase 2 (20 min/session), but from weeks 12 to 16 it will be increased by 5 min (i.e., until 25 min/session) with intensity from 75 to 85% of HRR. We will estimate intensity (% of HRR) by monitoring all sessions with Polar heart rate monitors. Moreover, the CR-10 Borg scale [45, 46] (values ranging from 0 to 10) will be used to evaluate perceived exertion during the aerobic part (range between 7 to 9 corresponding to 75 to 85% of HRR) and after that in order to control the final intensity in this part.

Whenever the aerobic target intensity (% of HRR) is not reached by means of speed increase (without running), the inclination of the treadmill will be increased. A previous study conducted in moderately obese adults (mean BMI 33.4 kg/m^2^) demonstrated that walking at slower speeds and on moderate inclines lowered the net muscle moments of the joints of the lower extremities [47]. Furthermore, following recommendations from this study, in the EFIBAR RCT, the treadmill inclination will be increased by a maximum of 1° every 2 weeks, and the final inclination will not exceed 6° to minimise the risk of the onset of chronic tibia pain [48].

### Programme adherence

Adherence to the exercise programme will be measured throughout the whole intervention period using a comprehensive tally sheet to be completed daily by the PT during and after each training session. The detailed tally sheet is shown in Additional file 3: Table S3. It describes measurement and reporting of adherence to the exercise programme (Item 5), the type and number of adverse events that occur during exercise (Item 11), the adherence and fidelity to the programme (Item 16a and 16b). Moreover, extra physical activity and/or exercise performed (Item 9) will be register as it has been recommended [46, 47]. Briefly, training attendance will be defined as the number of sessions attended divided the number of sessions prescribed (*n* = 48). Performing at least 80% of all planned training sessions will be considered a successful attendance rate. Additionally, self-efficacy and intention to be physically active will be measured at baseline (pre-surgery), post-intervention (4 months after surgery) and at 1-year follow-up examinations, due to their associations with adherence to the exercise programmes [48]. For self-efficacy, we will use a modification of the McAuley’s Exercise Self Efficacy Scale [48], whereas for measuring the intention to be physically active, we will adopt the question used by González-Cutre et al. [49]. During each training session, other variables related to adherence to the exercise programme will be collected, such as punctuality, extra physical activity, compliant attitude, and the Rate of Perceived Exertion for aerobic and strength training (Borg Scale RPE: 0–10 and ONMI resistance scale: 0–10, respectively). Heart rate (using a Polar V800 pulsometer) will be registered immediately after each part of the training session. Mood through Feeling Scale, Exercise-Induced Acute Onset Exhaustion by HPHEE scale [50] and the Rate of Perceived Exertion by Borg Scale (RPE 0–10) will be also measured before and after training sessions. All of these variables are presented in Additional file 3: Table S3. Another adherence strategy designed to maintain motivation throughout the intervention consists of WhatsApp motivational messages and videos (Item 6), which will be sent to programme participants; participants will receive WhatsApp messages every Friday and videos every month. The 16 motivational messages and four videos are shown in Table 2.

**Table 2:** Training periodization (Item 13)

Weeks		1			2			3			4			5			6			7			8			9			10			11			12			13			14			15			16		
*Session*		*1*	*2*	*3*	*4*	*5*	*6*	*7*	*8*	*9*	*10*	*11*	*12*	*13*	*14*	*15*	*16*	*17*	*18*	*19*	*20*	*21*	*22*	*23*	*24*	*25*	*26*	*27*	*28*	*29*	*30*	*31*	*32*	*33*	*34*	*35*	*36*	*37*	*38*	*39*	*40*	*41*	*42*	*43*	*44*	*45*	*46*	*47*	*48*
*Phases*		Familiarisation Phase												Phase 1												Phase 2						Phase 3																	
Warm-up	Aerobic activity	5 min of low intensity aerobic activity (50%-65% HRR) on Treadmill																																															
Strength training	Type of exercises performed	Learning exercises of movement patterns						Learning exercises of movement patterns and Weight-bearing and strength training with elastic bands						^c^Compensatory training: - Core stability - Stabiliser muscles (Level A-B)												^c^Compensatory training: - Core stability - Stabiliser muscles (Level A-B)						^c^Compensatory training: - Core stability - Stabiliser muscles (Level A-B-C)																	
								Compensatory training: - Core stability - Stabiliser muscles (Level A)						Exercises localised in major muscle groups												Exercises localised in major muscle groups						Exercises localised in major muscle groups																	
	Training stimulus aim	Initial adaptations to strength training												Metabolic stress												Metabolic stress/Mechanical tension and muscle damage						Mechanical tension and muscle damage																	
	Sets	3						Exercises of movement patterns, weight-bearing and elastic bands (2 set/5-7 rep) Exercise intensity: Thera-Band RPE scale						1	1	1	1	1	1	2	2	2	2	2	1	3	3	3	3	3	3	3	3	3	3	3	3	3	3	3	3	3	3	3	3	3	3	3	3
	Repetition	8-10												12	12	12	12	12	12	12	12	12	12	12	12	12	12	12	12	12	12	10	10	10	10	10	10	8	8	8	8	8	8	6	6	6	6	6	6
	Exercise Intensity^a^ (RM)	Weight-bearing												24	24	24	24	24	24	24	24	24	24	24	24	24	24	24	24	24	24	20	20	20	20	20	20	16	16	16	14	14	14	12	12	12	10	10	10
	(CE)^b^													12	12	12	12	12	12	12	12	12	12	12	12	12	12	12	12	12	12	10	10	10	10	10	10	8	8	8	6	6	6	6	6	6	4	4	4
	Total Rep													12	12	12	12	12	12	24	24	24	24	24	12	36	36	36	36	36	36	30	30	30	30	30	30	24	24	24	24	24	24	18	18	18	18	18	18
Aerobic training	Type/number of exercises performed	Treadmill			Treadmill			Treadmill			Treadmill			Treadmill			Treadmill			Treadmill			Treadmill			Treadmill			Treadmill			Treadmill			Treadmill			Treadmill			Treadmill			Treadmill			Treadmill		
	Aerobic training volume (min)	15	15	15	15	15	15	15	15	15	15	15	15	20	20	20	20	20	20	20	20	20	20	20	20	20	20	20	20	20	20	20	20	20	25	25	25	25	25	25	25	25	25	25	25	25	25	25	25
	Intensity (%HRR)	≤65%HRR												65-70%HRR												70-75%HRR						75-85%HRR																	
Cool-down	Flexibility	5 min static and dynamic flexibility exercises																																															

^a^*RM* Repetition Maximum. The weight that can be lifted “X” times before repetition failure

^b^*CE* Number of repetitions that could be performed and not performed with this weight

^c^Compensatory training: isometric exercises (20-30 seconds) and isokinetic movements (5-7 reps)

## Discussion

The EFIBAR RCT aims to investigate the effect of a 16-week supervised exercise programme on weight loss, body composition, cardiometabolic risk, physical fitness and quality of life in morbidly obese patients following BS. Physical exercise in the clinical setting has increased its presence due to the numerous benefits reported in a wide range of patients [51]. However, the description of exercise-based interventions in clinical trials has largely been poor [52]. CERT was recently developed as a tool to enhance replicability of exercise interventions.

To the best of our knowledge, this is the first evidence-based description of an exercise programme in morbid obese individuals based on CERT methodology. However, it should be noted that previous results in BS and obese patients have been considered and incorporated into the design of the present programme. For instance, Huck et al. [53] found that supervised strength training using free weights, body weight, or resistance bands safely improved muscular strength and physical functioning, increasing the patient’s capacity to perform daily activities after BS. In this study, each training session comprised 8–10 exercises targeting all major muscle groups, although the specific exercises performed were not reported. Castello-Simoes et al. [30] implemented diaphragmatic breathing to prepare the body for the subsequent activity. Colato et al. [54] employed a concurrent training programme using stack-weight equipment and concluded that an exercise training programme in the first 4 months is effective for improving body composition in overweight and obese patients (BMI 25–39.9 kg/m^2^). Consequently, in the current study, participants will start to train as soon as possible (1–2 weeks after BS), although the first increase in strength/aerobic intensity and volume will not occur until 1 month after surgery. Thus, we have incorporated previous knowledge into the design of this evidence-based intervention.

It should be recognised that some criteria are derived from previous studies carried out in non-morbidly obese (or BS) individuals, since the evidence in BS individuals is scarce. For example, the concurrent training order (i.e., strength before aerobic training) was established following studies in obese (but not BS) populations, in which this order was the most beneficial for improving body composition, physical fitness and other health biomarkers [54]. The structure of the exercise programme is the same as that of Colato et al. [54] in which 14 overweight and obese adults carried out 60 min training sessions, each including three parts: 1) warm-up exercises; 2) concurrent training; and 3) stretching. In our study, even while following the same structure, the intensity and training volume will adjust and progressively increase throughout the 16 weeks. In addition, we are aware that starting the training program 7–14 days after BS can be quite early for this patients. However, participants will go through a familiarisation period during the first 4 weeks, which only includes walking at low-moderate intensity and some exercises aimed at learning movement patterns, as well as exercises with a low speed and a high body control for a better body posture. Also, participants will follow a gradual progression to reach the assigned exercise dose. Additionally, our detailed tally sheet (Additional file 3: Table S3) will register the Exercise-Induced Acute Onset Exhaustion by HPHEE scale, the Rate of Perceived Exertion by Borg Scale (RPE 0–10) before and after each training session, as well as possible adverse events that may occur during exercise.

In this evidence-based exercise programme for BS patients, the exercise techniques and their rationale are described in detail, together with advice to carry them out appropriately. This ensures understanding by different health professionals from multidisciplinary teams, although the exercise professionals (i.e., well-qualified PTs) should play a relevant role in its implementation [55]. Exercise progressions have been designed such that the participants (and their PTs) can choose to increase or reduce the exercise difficulty (i.e., Level A, B or C) to allow all BS patients to undertake the programme. The main strength exercises are common and easy to perform, in order to be replicable in other contexts (e.g., clinical settings, fitness centers, research laboratories). Moreover, this programme includes a large number of body weight exercises that can be performed without any equipment, increasing its applicability in other studies or contexts.

Adherence to an exercise programme is fundamental to understanding the clinical efficacy of exercise-based interventions [56]. Consequently, we have carefully revised the BS literature with special attention to adherence strategies for exercise programmes. Wiklund and colleagues [57] conducted semi-structured interviews with 18 patients awaiting bariatric surgery. They reported that these patients tend to be uncomfortable appearing in public wearing exercise clothing and that exercising with someone at the same level of health/fitness increased motivation [57]. Therefore, to avoids patients' discomfort and to increase safety levels, we selected individual sessions (i.e., ratio 1:1), in which the PTs will monitor the level of adherence using a comprehensive tally sheet (Additional file 3: Table S3) during and after each training session. Furthermore, it is expected that the motivational WhatsApp and video messages will maintain patients’ motivation and confidence throughout the intervention. Accordingly, previous literature has demonstrated that health club members whose training is directed by well-qualified PTs administering evidence-based training regimens achieve significantly greater improvements in lean body mass and other dimensions of fitness than members who direct their own training. [55]. Thus, the strategies have been designed to maximise adherence and efficacy of the EFIBAR exercise programme.

Knowing that some of the exercises had not been previously carried out in the literature, the EFIBAR RCT included a 2-week pilot programme before start recruiting participants, during which three patients with severe obesity (two women and one man) participated. These patients had different conditions, permitting observation of responses to the exercise programme from different points of view (i.e., a patient who did not expect surgery, a patient waiting for surgery and a patient who had undergone surgery 1 week earlier). The three patients tested the exercise progression for 2 weeks. Accordingly, some of the initially prescribed exercises were modified. For instance, some of the proposed progressions were changed, and other proposed exercises were simply removed (e.g., due to patients’ discomfort). Finally, others have described that it is difficult to assess the appropriate type and amount of physical activity and/or exercise for BS patients, and it is unclear if preoperative exercise has the same effect as postoperative exercise [12]. Thus, it should be noted that the EFIBAR RCT exercise programme was carefully designed to be carried out after surgery, although we believe that it could also be conducted as a preoperative exercise programme.

Altogether, this report presents an evidence-based exercise intervention, following the CERT reporting guidelines, and a comprehensive rationale underlying each step conducted during its design. Consequently, this evidence-based report can not only be used for exercise prescription for morbidly obese individuals, but can also inform the use of the CERT guidelines for the comprehensive and transparent reporting of exercise interventions in BS patients and for patients with other diseases.

The present study details the exercise programme of the EFIBAR RCT, which may serve: 1) exercise professionals who would like to implement an evidence-based exercise programme for BS patients, and 2) as an example of the application of the CERT criteria. To the best of our knowledge, this is the first report of an exercise protocol based on CERT methodology in BS patients.

The results of the EFIBAR study will be disseminated through a strategic dissemination plan. The strategy will use different channels to reach a large number of stakeholder groups and individuals, at the local, national and international level; this will include dissemination in academic media (ie, peer-reviewed journal articles, national and international conference presentations), social media (ie, Facebook, Twitter), print media (ie, newspaper), broadcast media (ie, radio, television), the internet (ie, links to study reports on the University of Almería website), electronic and postal mail (ie, posting of study findings to participants and stakeholders) and community/stakeholder engagement activities (ie, community forums, stakeholder meetings).

## Additional files

Additional file 1: **Table S1.** Exercise equipment for The EFIBAR study. (DOCX 2428 kb)

Additional file 2: **Table S2.** Detailed description of The EFIBAR exercises Training Programme. (DOCX 18742 kb)

Additional file 3: **Table S3**. Detailed tally sheet used to assess adherence to exercise programme (Item 5), type and number of adverse events that occur during exercise (Item 11), and fidelity (Item 16a and 16b). (DOCX 29 kb)

## Data Availability

The clinical datasets will be available according to the terms established in the Clinicaltrials.gov NCT03497546, i.e., once the study ends and the main results are published, contacting the responsible party (artero@ual.es).

## Abbreviations

BS: Bariatric surgery
CERT: Consensus on Exercise Reporting Template
HRmax: Maximum real heart rate
HRR: Heart rate reserve
HRV: Heart rate variability
MET: Metabolic equivalent
PT: Personal Trainers
RCT: Randomised controlled trials
RM: Repetition maximum
RPE: Rating of Perceived Exertion
