# Supplementary material for: Supervised exercise following bariatric surgery in morbid obese adults: CERT-based exercise study protocol of the EFIBAR randomised controlled trial
Source: BMC Surg. 2019 Sep 5;19:127. doi: 10.1186/s12893-019-0566-9 (PMC6729089; doi:10.1186/s12893-019-0566-9)
Supplement: Supplementary file 1 — Table S1. Exercise equipment for The EFIBAR study. (DOCX 2428 kb) [file 12893_2019_566_MOESM1_ESM.docx]

**Additional file 1: Table S1** Exercise equipment for The EFIBAR study.

| **Equipment(Brand)** | **Number** | **Characteristics** | **Photo** |
| --- | --- | --- | --- |
| Spalier  (Azafit) | 2 | -Colour: Black. | 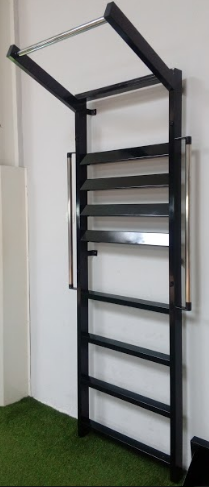 |
| Fitness mat  (Salter) | 9 | -Colour: Blue | 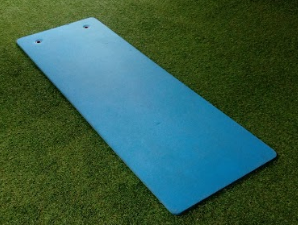 |
| Bosu  (Amaya sport) | 3 | -Colour: Blue | 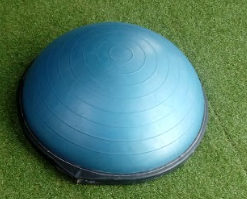 |
| Box  (Azafit) | 1 | -Colour: Black.  -Measurements: 60 x 50 x 40 cm | 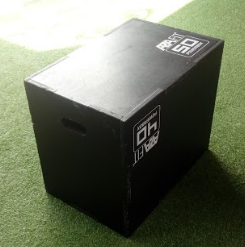 |
| Medicine Ball  (Azafit) | 3 | -Colour: Black and Grey.  -Weight: 3/6/9 kg | 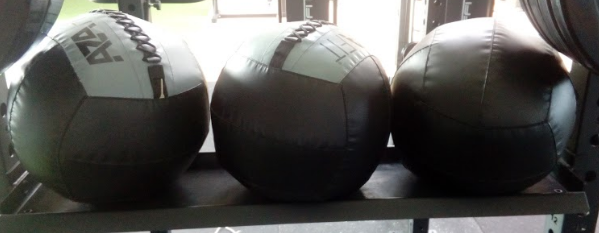 |
| Rack  (Azafit) | 1 | -Colour: Black.  -Supports: Adjustable supports to hold the bar in the rack and safety brackets for the cage. | 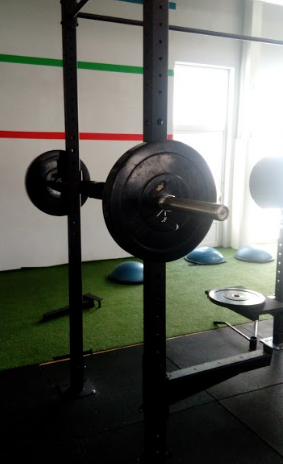 |
| Fitball  (Amaya sport) | 1 | -Colour: Grey.  -Diameter: 65 cm. | 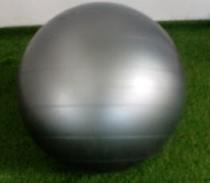 |
| Fitness bar  (Azafit) | 3 | -Colour: Grey.  -Weight: 20 kg. | 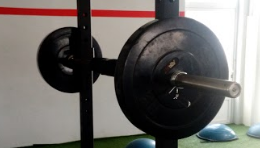 |
| Iron Bumper plate (Azafit) | 20 | -Colour: Black.  -Weight: 20/10/5 kg | 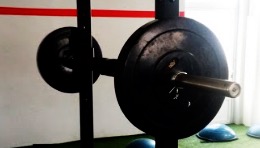 |
| Multipower  (Ffittech) | 2 | -Colour: Black.  -Types of grip: Open bar, closed grip and the rope grip. | 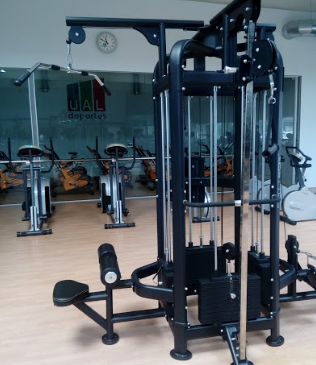 |
| Treadmill  (Salter) | 4 | Model: M836 (2) | 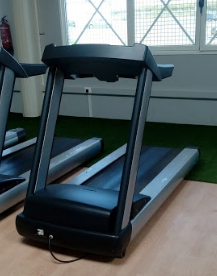 |
| Stationary bicycle  (Salter) | 3 | Model: M353 | 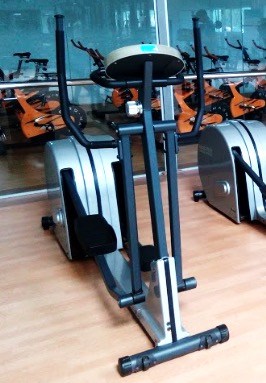 |
| Multipower  (Ffittech) | 2 | -Colour: Black.  -Types of grip: Open bar, closed grip and the rope grip. | 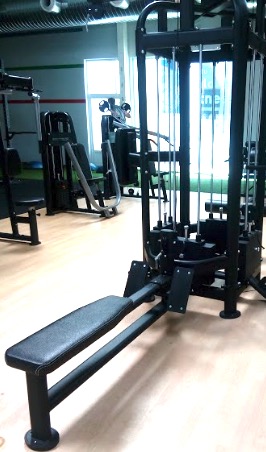 |
| Banks  (Ffittech) | 3 | -Colour: Black.  -Postions: 5 inclinations. | 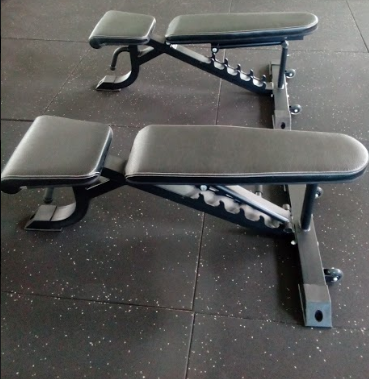 |
| Kettlebells  (Azafit) | 5 | -Colour: Black  -Weight: 4/ 8/ 12/ 18/ 20 kg | 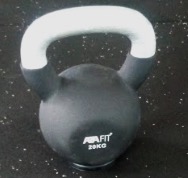 |
| Dumbbells  (Azafit) | 16 pairs | -Colour: Black.  -Weight: 1/ 2/ 3/ 4/ 5/ 7.5/ 10/ 12.5/ 15/ 17.5/ 20/ 22.5/ 25 kg | 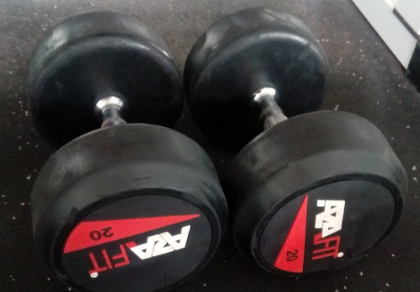 |
| Dumbbells  Salter | 5 pairs | -Colour: Black.  -With bearings.  -Weight: 26/ 27/ 28/ 29/ 30 kg | 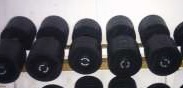 |
| Bars  (Salter) | 21 | -Colour: Grey.  -Weight: 2.15 kg | 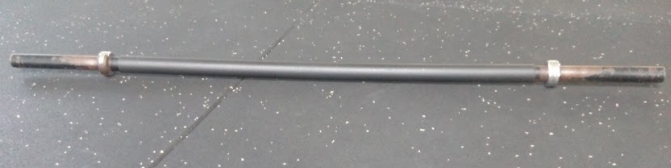 |
| Bars  (Salter) | 3 | -Colour: Grey.  -Weight: 10 kg. | 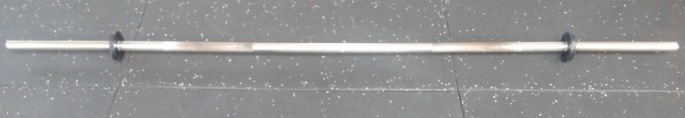 |
| Bumper Plate  (Salter) | 44 | -Colour: Black.  -Weight: 1.25/ 2.5/ 5/ 10/ 15/ 20 | 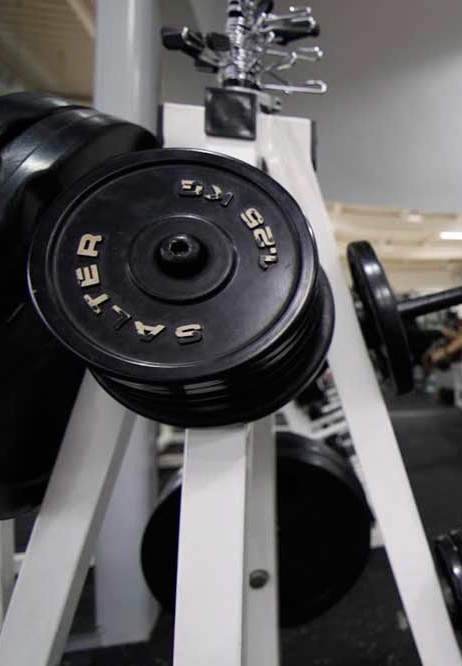 |
| Resistance Bands  (AFW) | 2 | -Yellow: Low resistance.  -Green: Medium resistance.  -Blue: High resistance. | 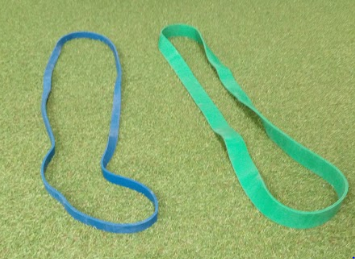 |
| Therabands  (AFW) | 3 | -Yellow: Low resistance.  -Green: High resistance. | 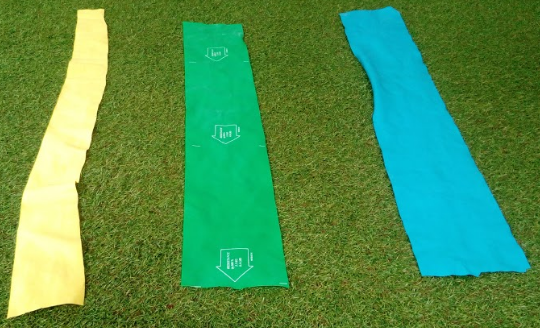 |
