# Supplementary material for: Supervised exercise following bariatric surgery in morbid obese adults: CERT-based exercise study protocol of the EFIBAR randomised controlled trial
Source: BMC Surg. 2019 Sep 5;19:127. doi: 10.1186/s12893-019-0566-9 (PMC6729089; doi:10.1186/s12893-019-0566-9)
Supplement: Supplementary file 2 — Table S2. Detailed description of The EFIBAR exercises Training Programme. (DOCX 18742 kb) [file 12893_2019_566_MOESM2_ESM.docx]

**Additional file 2: Table S2** Detailed description of The EFIBAR exercises Training Programme.

| **EFIBAR EXERCISES TRAINING PROGRAMME** | | | | | | | | |
| --- | --- | --- | --- | --- | --- | --- | --- | --- |
| **Learning of movement patterns** | | | | | | | | |
| **1. Breathing** | | | | | | | | |
|  | | | | | | | | |
| **LEVEL A** |  | **VARIANT 1** |  | **VARIANT 2** |  | **VARIANT 3** | |  |
| **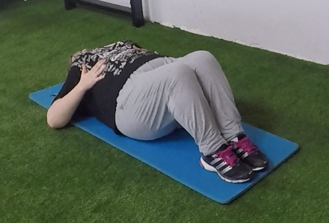**  Recommended from the second-third week |  | **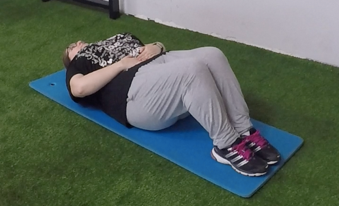** |  | **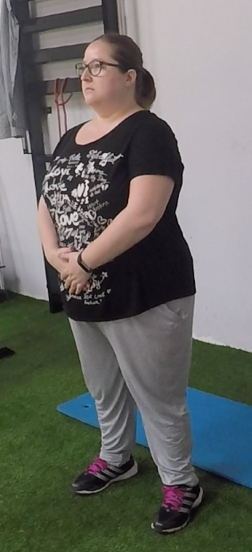** |  | **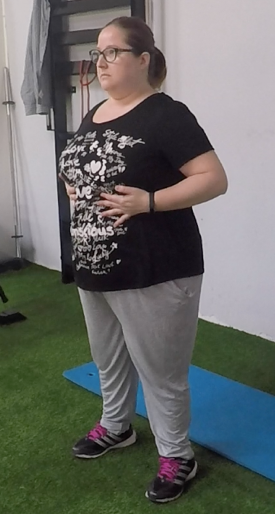** | |  |
|  |  |  |  |  |  |  |  |  |
|  |  |  |  |  |  |  |  |  |
|  |  |  |  |  |  |  |  |  |
|  |  |  |  |  |  |  |  |  |
|  |  |  |  |  |  |  |  |  |
|  |  |  |  |  |  |  |  |  |
|  |  |  |  |  |  |  |  |  |
|  |  |  |  |  |  |  |  |  |
|  |  |  |  |  |  |  |  |  |
| **Exercise Description:** | | | | | | | | |
| **LEVEL A** | | | | | | | | |
| Lying supine Thoracic Breathing:   - Initial Position: Lying supine with legs bent, feet flat on the floor and hands on the rib cage. - Exercise: Take a deep breath, noticing how the chest expands and expires, releasing all the air slowly through the mouth. | | | | | | | | |
|  |  |  |  |  |  |  |  |  |
|  |  |  |  |  |  |  |  |  |
|  |  |  |  |  |  |  |  |  |
| **VARIANT 1** | | | | | | | | |
| Lying supine Abdominal Breathing:   - Initial Position: Lying supine with legs bent, feet flat on the floor and hands on the abdomen. - Exercise: Take a deep breath, noticing how the abdominal area expands and expires, releasing all the air slowly through the mouth. | | | | | | | | |
|  |  |  |  |  |  |  |  |  |
|  |  |  |  |  |  |  |  |  |
|  |  |  |  |  |  |  |  |  |
| **VARIANT 2** | | | | | | | | |
| Thoracic Standing breathing:   - Initial Position: Stand with hands on the rib cage. - Exercise: Take a deep breath, noticing how chest expands and expires, releasing all the air slowly through the mouth. | | | | | | | | |
|  |  |  |  |  |  |  |  |  |
|  |  |  |  |  |  |  |  |  |
|  |  |  |  |  |  |  |  |  |
| **VARIANT 3** | | | | | | | | |
| Abdominal Standing breathing:   - Initial Position: Stand with hands on the abdomen. - Exercise: Take a deep breath, noticing how the abdominal area and expires, releasing all the air slowly through the mouth. | | | | | | | | |
|  |  |  |  |  |  |  |  |  |
|  |  |  |  |  |  |  |  |  |
|  |  |  |  |  |  |  |  |  |
| **Learning of movement patterns** | | | | | | |  |  |
| **2. Lying Down Lumbopelvic Dissociation** | | | | | | |  |  |
|  | | | | | | |  |  |
| **LEVEL A** |  | **VARIANT 1** |  | | | |  |  |
| **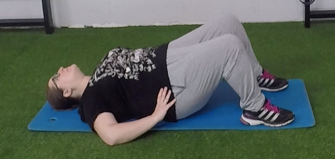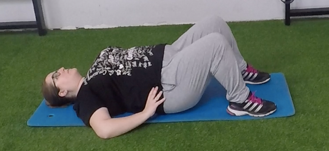**  Recommended from the second-third week |  | **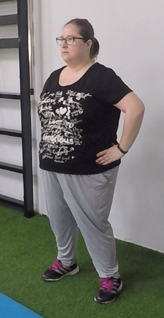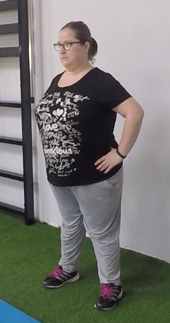** |  |  |  |  |  |  |
|  |  |  |  |  |  |  |  |  |
|  |  |  |  |  |  |  |  |  |
|  |  |  |  |  |  |  |  |  |
|  |  |  |  |  |  |  |  |  |
|  |  |  |  |  |  |  |  |  |
|  |  |  |  |  |  |  |  |  |
|  |  |  |  |  |  |  |  |  |
|  |  |  |  |  |  |  |  |  |
|  |  |  |  |  |  |  |  |  |
| **Exercise Descriptions:** | | | | | | |  |  |
| **LEVEL A** | | | | | | |  |  |
| Lying supine Lumbopelvic Dissociation:   - Initial Position: Lying supine with legs bent and feet flat on the floor. - Exercise: Consciously perform retroversion and anteversion of the hip. | | | | | | |  |  |
|  |  |  |  |  |  |  |  |  |
|  |  |  |  |  |  |  |  |  |
|  |  |  |  |  |  |  |  |  |
| **VARIANT 1** | | | | | | |  |  |
| Standing Lumbopelvic Dissociation:   - Initial Position: Stand with hands on the hip. - Exercise: Consciously perform retroversion and anteversion of the hip. | | | | | | |  |  |
|  |  |  |  |  |  |  |  |  |
|  |  |  |  |  |  |  |  |  |
|  |  |  |  |  |  |  |  |  |

| **Learning of movement patterns** | | | |
| --- | --- | --- | --- |
| **3. Dorsal-Lumbar Mobilization (Cat Camel)** | | | |
|  | | | |
| **LEVEL A** |  | **VARIANT 1** |  |
| **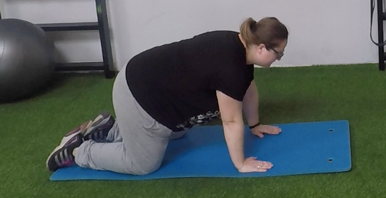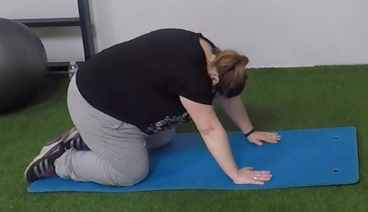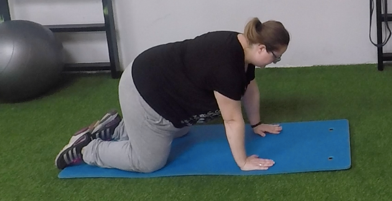** |  | **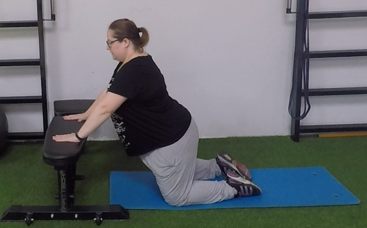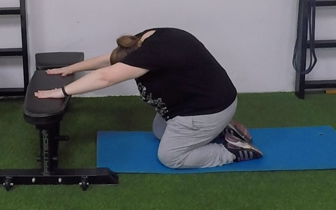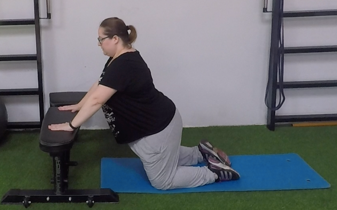** |  |
|  |  |  |  |
|  |  |  |  |
|  |  |  |  |
|  |  |  |  |
|  |  |  |  |
|  |  |  |  |
|  |  |  |  |
|  |  |  |  |
|  |  |  |  |
| **Exercise Descriptions:** | | | |
| **LEVEL A** | | | |
| Cat-camel Lying prone in quadruped on the floor.   - Initial Position: With back erect, head and hip in neutral position, the hip flexed. Support the hands and knees on the floor. - Exercise:   *First movement.* Perform a trunk flexion with hip anteversion and neck flexion.  *Second movement.* Perform a small trunk and neck hyperextension, in addition to a hip anteversion. | | | |
|  |  |  |  |
|  |  |  |  |
|  |  |  |  |
| **VARIANT 1** | | | |
| Cat-camel leaning on a bench.   - Initial Position: With back erect, head and hip in neutral position, the hip slightly flexed. Support the hands on a bench with low high and knees on the floor - Exercise:   *First movement.* Perform a trunk flexion with hip anteversion and neck flexion.  *Second movement.* Perform a small trunk and neck hyperextension, in addition to a hip anteversion. | | | |
|  |  |  |  |
|  |  |  |  |
|  |  |  |  |

| **Learning of movement patterns** | | | | | |
| --- | --- | --- | --- | --- | --- |
| **4. Middle-squat** | | | | | |
|  | | | | | |
| **LEVEL A** |  | **VARIANT 1** |  | **VARIANT 2** |  |
| **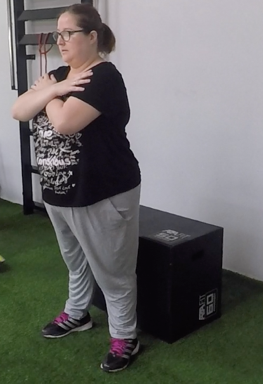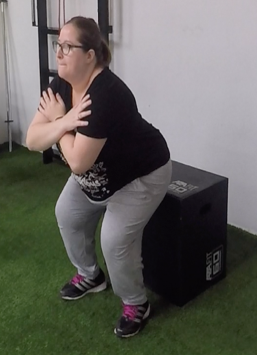** |  | **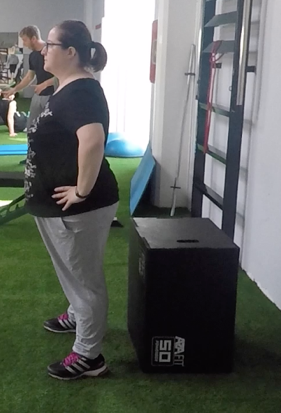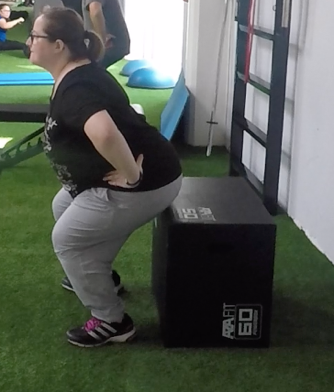** |  | **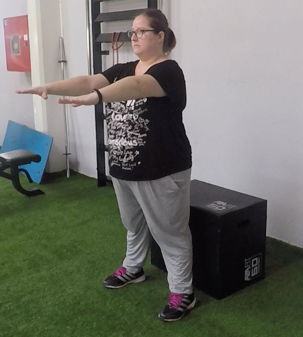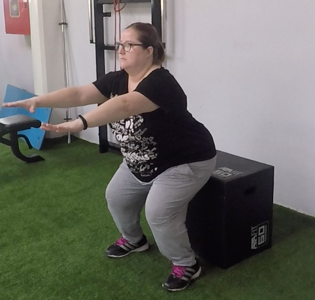** |  |
|  |  |  |  |  |  |
|  |  |  |  |  |  |
|  |  |  |  |  |  |
|  |  |  |  |  |  |
|  |  |  |  |  |  |
|  |  |  |  |  |  |
|  |  |  |  |  |  |
|  |  |  |  |  |  |
|  |  |  |  |  |  |
| **Exercise Description:** | | | | | |
| **LEVEL A** | | | | | |
| Sit on the chair with crossed arms in the chest:   - Initial Position: Stand with your feet at shoulder width, or slightly wider and the toes pointed slightly outward and crossed arms in the chest. - Exercise:   *Eccentric phase.* With the back erect and the hip in a neutral position, a lowering of the body is performed by a triple flexion: hip, knee and ankle until getting to touch the chair. The movement should be performed by moving the hips backwards, thus preventing the knees from advancing too far ahead of the tip of the feet.  *Concentric phase.* Return to the starting position in the same way as in the eccentric phase. | | | | | |
|  |  |  |  |  |  |
|  |  |  |  |  |  |
|  |  |  |  |  |  |
| **VARIANT 1** | | | | | |
| Sit on the chair with hands on hips:   - Initial Position: Stand with your feet at shoulder width, or slightly wider and the toes pointed slightly outward and hands on hip. - Exercise:   *Eccentric phase.* With the back erect and the hip in a neutral position, a lowering of the body is performed by a triple flexion: hip, knee and ankle until getting to touch the chair. The movement should be performed by moving the hips backwards, thus preventing the knees from advancing too far ahead of the tip of the feet.  *Concentric phase.* Return to the starting position in the same way as in the eccentric phase. | | | | | |
|  |  |  |  |  |  |
|  |  |  |  |  |  |
|  |  |  |  |  |  |
| **VARIANT 2** | | | | | |
| Sit on the chair with outstretched arms:   - Initial Position: Stand with your feet at shoulder width, or slightly wider and the toes pointed slightly outward and outstretched arms. - Exercise:   *Eccentric phase.* With the back erect and the hip in a neutral position, a lowering of the body is performed by triple flexion: hip, knee and ankle until getting to touch the chair. The movement should be performed by moving the hips backwards, thus preventing the knees from advancing too far ahead of the tip of the feet.  *Concentric phase.* Return to the starting position in the same way as in the eccentric phase. | | | | | |
|  |  |  |  |  |  |
|  |  |  |  |  |  |
|  |  |  |  |  |  |
|  |  |  |  |  |  |
|  |  |  |  |  |  |
|  |  |  |  |  |  |

| **Learning of movement patterns** | |
| --- | --- |
| **5. Standing Up Lumbopelvic Dissociation** | |
|  | |
| **LEVEL A** |  |
| **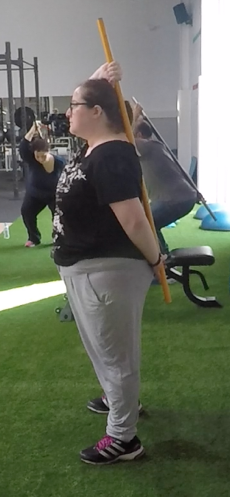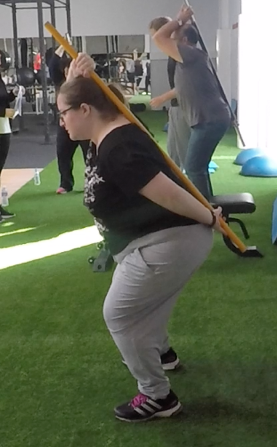** |  |
|  |  |
|  |  |
|  |  |
|  |  |
|  |  |
|  |  |
|  |  |
|  |  |
|  |  |
| **Exercise Descriptions:** | |
| **LEVEL A** | |
| Lumbopelvic Dissociation Standing (Progression trunk bar)   - Initial Position: Standing up straight facing forward, with a pike hold with one hand above the head and another below the lower back, the pike will be in contact with the head, back and lumbar area. - Exercise:   *Eccentric phase.* With the back erect, the hip and head in a neutral position, a lowering of the body is performed by a triple flexion: hip, knee and ankle. Always maintaining the contact of the 3 points with the pike.  *Concentric phase.* Returning to the starting position in the same way as in the eccentric phase. | |
|  |  |
|  |  |
|  |  |
|  |  |
|  |  |
|  |  |

| **Learning of movement patterns** | |
| --- | --- |
| **6. Deadlift** | |
|  | |
| **LEVEL A** |  |
| **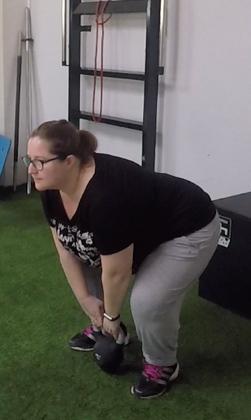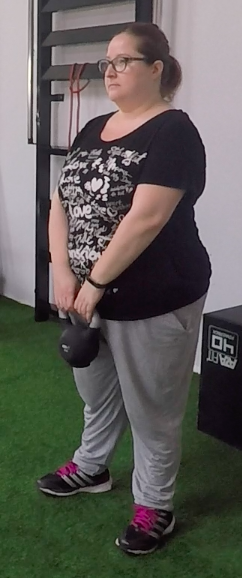**  Recommended from the third-fourth week |  |
|  |  |
|  |  |
|  |  |
|  |  |
|  |  |
|  |  |
|  |  |
|  |  |
|  |  |
| **Exercise Descriptions:** | |
| **LEVEL A** | |
| Deadlift (With weight within the center of gravity)   - Initial Position: Feet shoulder width with knees bent close to 90 °, hip flexion with trunk in neutral position and back straight. Dumbbell between the legs with arms stretched. - Exercise:   *Concentric phase.* Keeping the back straight, we extend the legs until the dumbbell reaches the knees and from there we accompany it with a hip extension until reaching the vertical position  *Eccentric phase.* Controlling the descent movement of the dumbbell, following the same path we return to the starting position. | |
|  |  |
|  |  |
|  |  |
|  |  |
|  |  |
|  |  |

| **Learning of movement patterns** | | | | | |
| --- | --- | --- | --- | --- | --- |
| **7. Overhead Semi-Squat** | | | | | |
|  | | | | | |
| **LEVEL A** |  | **LEVEL B** |  | **LEVEL C** |  |
| **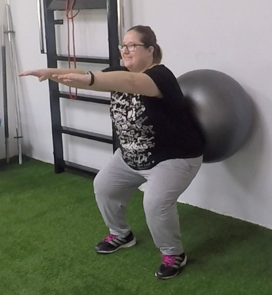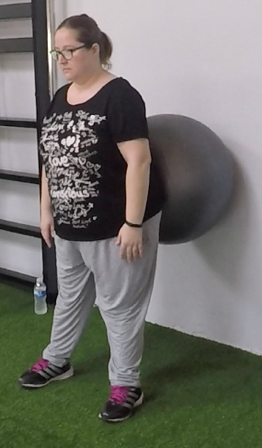** |  | **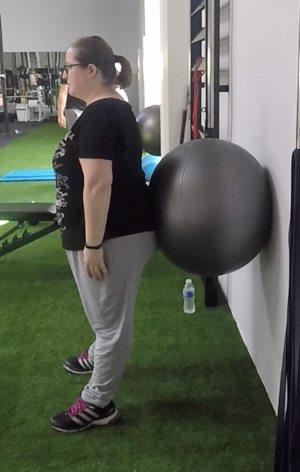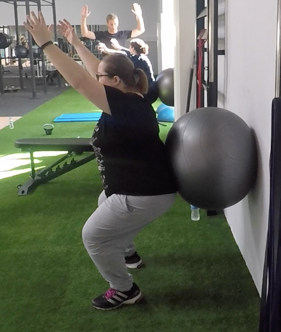** |  | **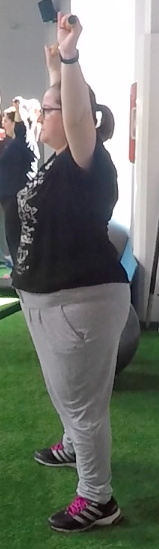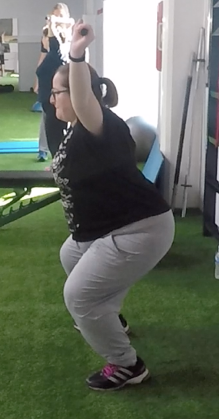** |  |
|  |  |  |  |  |  |
|  |  |  |  |  |  |
|  |  |  |  |  |  |
|  |  |  |  |  |  |
|  |  |  |  |  |  |
|  |  |  |  |  |  |
|  |  |  |  |  |  |
|  |  |  |  |  |  |
|  |  |  |  |  |  |
| **Exercise Descriptions:** | | | | | |
| **LEVEL A** | | | | | |
| Overhead Semi-Squat support in a fitball with arms above the shoulders   - Initial Position: Stand with your feet at shoulder width, or slightly wider and the toes pointed slightly outward, the lumbar area resting in a fit ball which will be supported in a wall. Arms above the shoulders. - Exercise:   *Eccentric phase*. Maintaining the arms position and the supported on the fit ball, perform a lowering of the body carrying a triple flexion: hip, knee and ankle.  *Concentric phase.* Returning to the starting position in the same way as in the eccentric phase. | | | | | |
|  |  |  |  |  |  |
|  |  |  |  |  |  |
|  |  |  |  |  |  |
| **LEVEL B** | | | | | |
| Overhead Semi-Squat supported in a fit ball with arms above the shoulders   - Initial Position: Stand with your feet at shoulder width, or slightly wider and the toes pointed slightly outward, the lumbar area resting in a fitball which will be supported in a wall. Arms above the shoulders. - Exercise:   *Eccentric phase.* Maintaining the arms position and the supported on the fit ball, perform a lowering of the body carrying a triple flexion: hip, knee and ankle.  *Concentric phase.* Returning to the starting position in the same way as in the eccentric phase. | | | | | |
|  |  |  |  |  |  |
|  |  |  |  |  |  |
|  |  |  |  |  |  |
| **LEVEL C** | | | | | |
| Overhead Semi-Squat supported in a fit ball with arms at shoulder height   - Initial Position: Stand with your feet at shoulder width, or slightly wider and the toes pointed slightly outward, the lumbar area resting in a fit ball which will be supported in a wall. Arms at shoulder height - Exercise:   *Eccentric phase.* Maintaining the arms position and the supported on the fit ball, perform a lowering of the body carrying a triple flexion: hip, knee and ankle.  *Concentric phase.* Returning to the starting position in the same way as in the eccentric phase. | | | | | |
|  |  |  |  |  |  |
|  |  |  |  |  |  |
|  |  |  |  |  |  |
|  |  |  |  |  |  |
|  |  |  |  |  |  |
|  |  |  |  |  |  |

| **Core and stabilisers exercises** | | | | | |
| --- | --- | --- | --- | --- | --- |
| **1. Frontal Plank (isometric)** | | | | | |
|  | | | | | |
| **LEVEL A** |  | **LEVEL B** |  | **LEVEL C** |  |
| 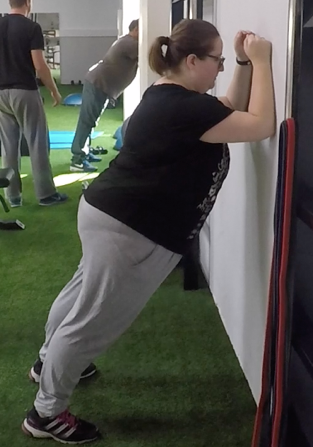 |  | 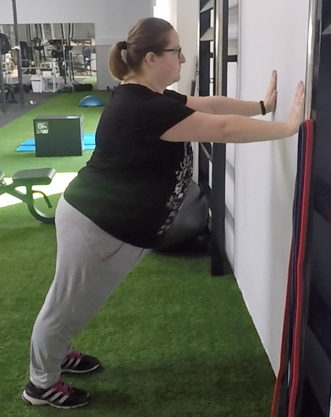 |  | 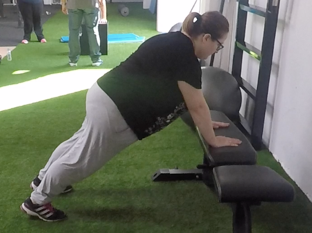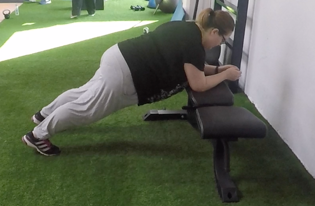 |  |
|  |  |  |  |  |  |
|  |  |  |  |  |  |
|  |  |  |  |  |  |
|  |  |  |  |  |  |
|  |  |  |  |  |  |
|  |  |  |  |  |  |
|  |  |  |  |  |  |
|  |  |  |  |  |  |
|  |  |  |  |  |  |
| **Exercise Descriptions:** | | | | | |
| **LEVEL A** | | | | | |
| **All variants: Back and hips should stay in neutral position throughout the exercise.  Frontal plank with elbows resting on the wall.   - Initial Position: Stand separated from the wall, tilted forward with arms bent and an elbow resting on the wall. - Exercise: The exercise will consist in keep the position. | | | | | |
|  |  |  |  |  |  |
|  |  |  |  |  |  |
|  |  |  |  |  |  |
| **LEVEL B** | | | | | |
| Frontal plank Supported on the wall with outstretched arms.   - Initial Position: Stand separated from the wall, tilted forward with arms outstretched and a hand resting on the wall. - Exercise: The exercise will consist in keep the position. | | | | | |
|  |  |  |  |  |  |
|  |  |  |  |  |  |
|  |  |  |  |  |  |
| **LEVEL C** | | | | | |
| Frontal plank leaning on a bench with elbows or arms stretched out.   - Initial Position: Lying supine supporting the tip of the feet in the floor and hands/elbows on a bench. - *Exercise*: The exercise will consist in keep the position. | | | | | |
|  |  |  |  |  |  |
|  |  |  |  |  |  |
|  |  |  |  |  |  |

| **Core and stabilisers exercises** | | | | | | | |
| --- | --- | --- | --- | --- | --- | --- | --- |
| **2. Side Plank (isometric)** | | | | | | | |
|  | | | | | | | |
| **LEVEL A** |  | **LEVEL B** |  | **LEVEL C** |  |  |  |
| 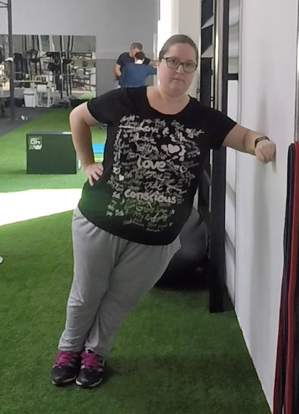 |  | 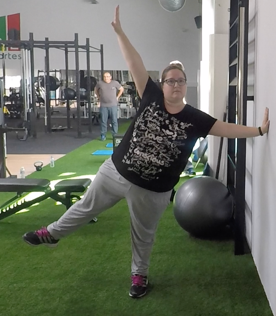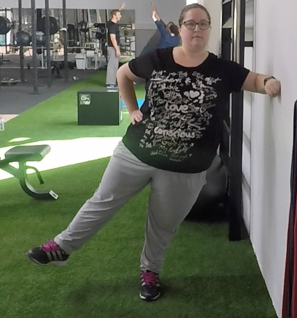 |  | 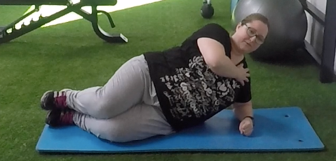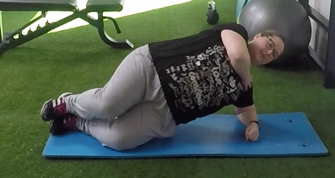 |  |  |  |
| **Exercise Descriptions:** | | | | | | | |
| **LEVEL A** | | | | | | | |
| **All variants: Back and hips should stay in neutral position throughout the exercise.  Side Lateral Plank with elbows resting on the wall.   - Initial Position: Stand separated from the wall, leaving a side the wall, tilted forward with arms bent and an elbow resting on the wall. - Exercise: The exercise will consist in keep the position. | | | | | | | |
|  |  |  |  |  |  |  |  |
|  |  |  |  |  |  |  |  |
|  |  |  |  |  |  |  |  |
| **LEVEL B** | | | | | | | |
| Side Lateral Plank Supported on the wall with outstretched arms   - Initial Position: Stand separated from the wall, leaving a side the wall, tilted forward with arms outstretched and hands resting on the wall. - Exercise: The exercise will consist in keep the position.   Variant: the same but doing an abduction of the opposite leg towards the side that we have supported on the wall.  Variant 2: the same but doing an abduction of the opposite leg towards the side that we have supported on the wall and lift over the shoulder the stretched arm of the same side. | | | | | | | |
|  |  |  |  |  |  |  |  |
|  |  |  |  |  |  |  |  |
|  |  |  |  |  |  |  |  |
| **LEVEL C** | | | | | | | |
| Side Lateral Plank lying down supported with elbows.   - Initial Position: Lying on the side, supporting the elbow on the floor and on one knee. - Exercise: The exercise will consist in keep the position. | | | | | | | |
|  |  |  |  |  |  |  |  |
|  |  |  |  |  |  |  |  |
|  |  |  |  |  |  |  |  |

| **Core and stabilisers exercises** | | | | | |
| --- | --- | --- | --- | --- | --- |
| **3. Push-up (isometric)** | | | | | |
|  | | | | | |
| **LEVEL A** |  | **LEVEL B** |  | **LEVEL C** |  |
| 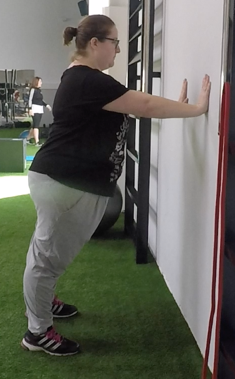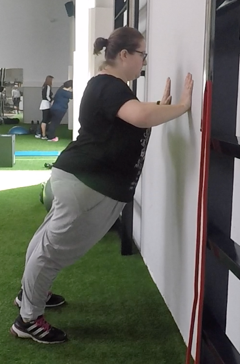 |  | 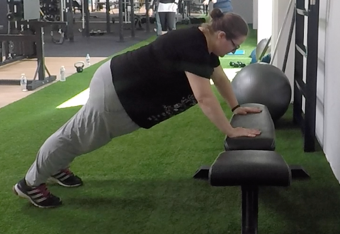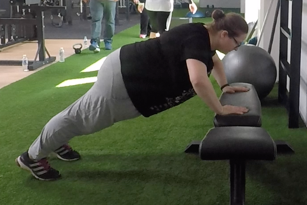 |  | 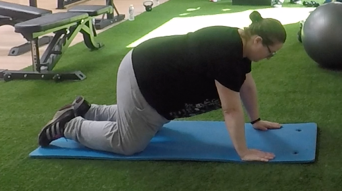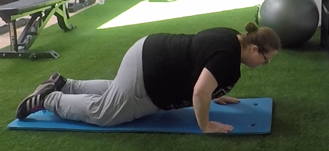 |  |
|  |  |  |  |  |  |
|  |  |  |  |  |  |
|  |  |  |  |  |  |
|  |  |  |  |  |  |
|  |  |  |  |  |  |
|  |  |  |  |  |  |
|  |  |  |  |  |  |
|  |  |  |  |  |  |
|  |  |  |  |  |  |
| **Exercise Descriptions:** | | | | | |
| **LEVEL A** | | | | | |
| **All variants: Back and hips should stay in neutral position throughout the exercise.  **All variants: It is recommended to make an approximation of the scapulae throughout the movement.  Push-up on the wall   - Initial Position: Stand separated from the wall, tilted forward with arms outstretched and hands resting on the wall, placed a little more open than the shoulders width. - Exercise:   *Eccentric phase.* Perform a lowering of the body, performing humeral abduction and elbow flexion in a controlled way until leaving the face a few centimetres from the wall.  *Concentric phase.* Push the body to the starting position. | | | | | |
|  |  |  |  |  |  |
|  |  |  |  |  |  |
|  |  |  |  |  |  |
| **LEVEL B** | | | | | |
| Push-up on a bench   - Initial Position: Lying supine with arms outstretched and hands resting on a bench, placed a little more open than the shoulders width. Feet supported on the floor. - Exercise:   *Eccentric phase.* Perform a lowering of the body, performing a 45º humeral abduction and a 90º elbow flexion in a controlled way until leaving the face a few centimetres from the bench.  *Concentric phase.* Push the body to the starting position. | | | | | |
|  |  |  |  |  |  |
|  |  |  |  |  |  |
|  |  |  |  |  |  |
| **LEVEL C** | | | | | |
| Push-up with knees supported   - Initial Position: Lying supine with arms outstretched and hands resting on the floor, placed a little more open than the shoulders width. Knees supported on the floor. - Exercise:   *Eccentric phase.* Perform a lowering of the body, performing a 45º humeral abduction and a 90º elbow flexion in a controlled way until leaving the face a few centimetres from the ground.  *Concentric phase.* Push the body to the starting position. | | | | | |
|  |  |  |  |  |  |
|  |  |  |  |  |  |
|  |  |  |  |  |  |
|  |  |  |  |  |  |
|  |  |  |  |  |  |
|  |  |  |  |  |  |

| **Core and stabilisers exercises** | | | | | |
| --- | --- | --- | --- | --- | --- |
| **4. Glute Bridge** | | | | | |
|  | | | | | |
| **LEVEL A** |  | **LEVEL B** |  | **LEVEL C** |  |
| 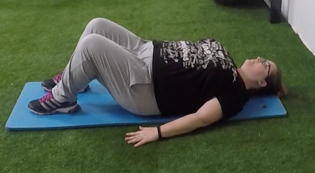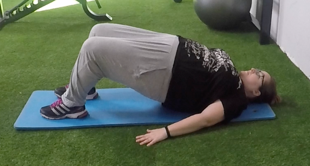 |  | 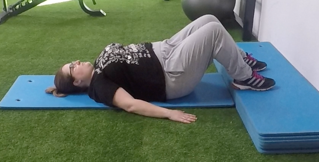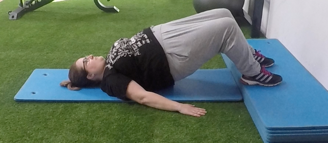 |  | 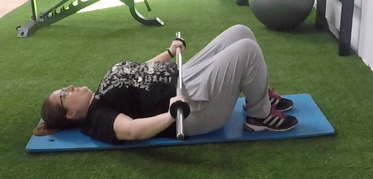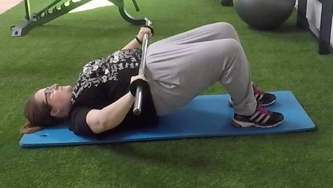 |  |
|  |  |  |  |  |  |
|  |  |  |  |  |  |
|  |  |  |  |  |  |
|  |  |  |  |  |  |
|  |  |  |  |  |  |
|  |  |  |  |  |  |
|  |  |  |  |  |  |
|  |  |  |  |  |  |
|  |  |  |  |  |  |
| **Exercise Description:** | | | | | |
| **LEVEL A** | | | | | |
| Gluteal Bridge   - Initial Position: Lying supine with legs bent, feet and hands supported on the floor. - Exercise:   *Concentric phase.* Make the extension of the hip until getting a straight line from the shoulder to the knees with neutral position of the hips and back.  *Eccentric phase.* Perform a controlled hip flex to reach ground. | | | | | |
|  |  |  |  |  |  |
|  |  |  |  |  |  |
|  |  |  |  |  |  |
| **LEVEL B** | | | | | |
| Gluteal Bridge with feet resting on high.   - Initial Position: Lying supine with legs bent, feet flat on high (a bench or a step) and hands supported on the floor. - Exercise:   *Concentric phase.* Make the extension of the hip until getting a straight line from the shoulder to the knees with neutral position of the hip.  Eccentric phase. Perform a controlled hip flex to reach ground. | | | | | |
|  |  |  |  |  |  |
|  |  |  |  |  |  |
|  |  |  |  |  |  |
| **LEVEL C** | | | | | |
| Gluteal Bridge with a Pike.   - Initial Position: Lying supine with legs bent, feet flat on the floor and hand on pike placed on the hip. - Exercise:   *Concentric phase.* Make the extension of the hip until getting a straight line from the shoulder to the knees with neutral position of the hip.  *Eccentric phase.* Perform a controlled hip flex to reach ground. | | | | | |
|  |  |  |  |  |  |
|  |  |  |  |  |  |
|  |  |  |  |  |  |

| **Core and stabilisers exercises** | | | | | | |  |
| --- | --- | --- | --- | --- | --- | --- | --- |
| **5. Bird-dogs** | | | | | | |  |
|  | | | | | | |  |
| **LEVEL A** |  | **LEVEL B** | |  | **LEVEL C** |  |  |
| 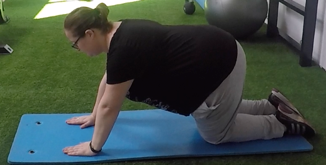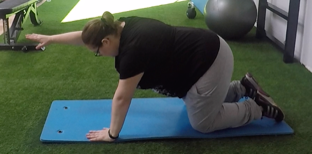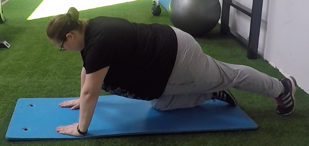 |  | 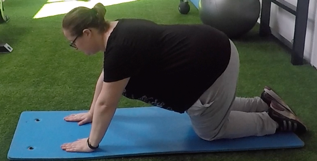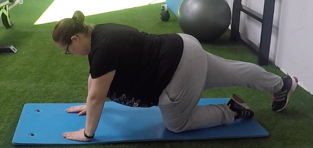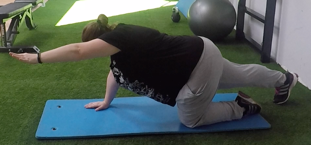 | |  | 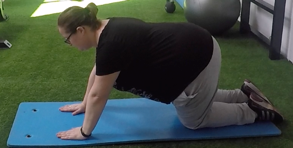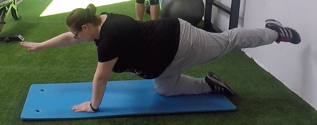 |  |  |
|  |  |  |  |  |  |  |  |
|  |  |  |  |  |  |  |  |
|  |  |  |  |  |  |  |  |
|  |  |  |  |  |  |  |  |
|  |  |  |  |  |  |  |  |
|  |  |  |  |  |  |  |  |
|  |  |  |  |  |  |  |  |
|  |  |  |  |  |  |  |  |
|  |  |  |  |  |  |  |  |
| **Exercise Descriptions:** | | | | | | |  |
| **LEVEL A** | | | | | | |  |
| **All variants: Back and hips should stay in neutral position throughout the exercise.  Bird-dogs Lifting leg or arm.   - Initial Position: Lying prone quadruped on knees and hands, with hip and back in neutral position. - Exercise:   *Concentric phase.* Perform a hip and knee extension until reaching the horizontal or perform a shoulder flexion until reaching the horizontal.  *Eccentric phase.* Carry out lowering of the leg or arm until returning to the initial position. | | | | | | |  |
|  |  |  |  |  |  |  |  |
|  |  |  |  |  |  |  |  |
|  |  |  |  |  |  |  |  |
| **LEVEL B** | | | | | | |  |
| Bird-dogs with low elevation:   - Initial Position: Lying prone quadruped on knees and hands, with hip and back in neutral position. - Exercise:   *Concentric phase.* Perform a hip and knee extension and a shoulder flexion until reaching 45º. Rise opposite arm and leg.  *Eccentric phase.* Carry out lowering of the leg and arm until returning to the initial position. | | | | | | |  |
|  |  |  |  |  |  |  |  |
|  |  |  |  |  |  |  |  |
|  |  |  |  |  |  |  |  |
| **LEVEL C** | | | | | | |  |
| Bird-dogs   - Initial Position: Lying prone quadruped on knees and hands, with hip and back in neutral position. - Exercise:   *Concentric phase.* Perform a hip and knee extension, and a shoulder flexion until reaching the horizontal. Rise opposite arm and leg.  *Eccentric phase.* Carry out lowering of the leg and arm until returning to the initial position | | | | | | |  |
|  |  |  |  |  |  |  |  |
|  |  |  |  |  |  |  |  |
|  |  |  |  |  |  |  |  |
| **Core and stabilisers exercises** | | | | | | | |
| **6. Crunch (modified McGill)** | | | | | | | |
|  | | | | | | | |
| **LEVEL A** | | |  | | | | |
| 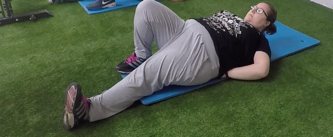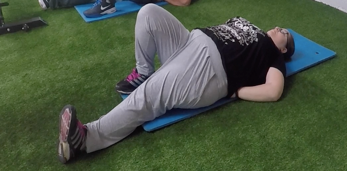 | | |  |  |  |  |  |
|  |  |  |  |  |  |  |  |
|  |  |  |  |  |  |  |  |
|  |  |  |  |  |  |  |  |
|  |  |  |  |  |  |  |  |
|  |  |  |  |  |  |  |  |
|  |  |  |  |  |  |  |  |
|  |  |  |  |  |  |  |  |
|  |  |  |  |  |  |  |  |
|  |  |  |  |  |  |  |  |
| **Exercise Descriptions:** | | | | | | | |
| **LEVEL A** | | | | | | | |
| McGill Crunch.   - Initial Position: Lying supine with a leg bent and the other extended, hands placed under lower back area. - Exercise:   *Concentric phase.* Maintaining the hip in neutral position, avoiding losing the lumbar curvature, perform a slightly trunk flexion, separating the head from the ground.  *Eccentric phase.* Back to the initial position, performing a trunk extension and supporting the head in the ground again. | | | | | | | |
|  |  |  |  |  |  |  |  |
|  |  |  |  |  |  |  |  |
|  |  |  |  |  |  |  |  |
|  |  |  |  |  |  |  |  |
|  |  |  |  |  |  |  |  |
|  |  |  |  |  |  |  |  |

| **Core and stabilisers exercises** | | | | | |
| --- | --- | --- | --- | --- | --- |
| **7. Press Palof** | | | | | |
|  | | | | | |
| **LEVEL A** |  | **LEVEL B** |  | 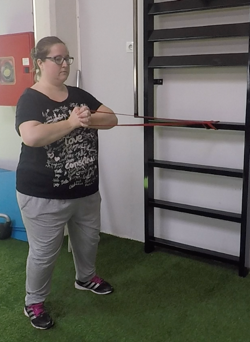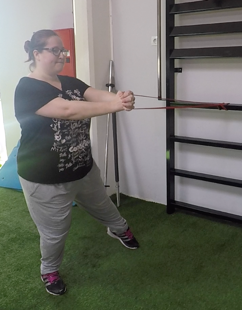  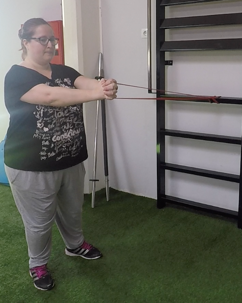 |  |
| 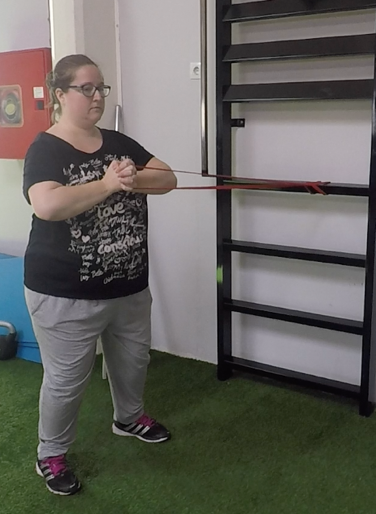 |  | 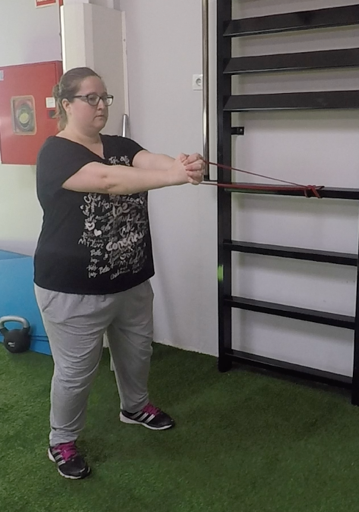 |  |  |  |
|  |  |  |  | **LEVEL C** |  |
| **Exercise Descriptions:** | | | | | |
| **LEVEL A** | | | | | |
| Isometric.   - Initial position: Standing up with arms outstretched and holding a band which will be extended and fastened on the spalier to one side of the subject. - Exercise: The exercise will consist in keeping the position. | | | | | |
|  |  |  |  |  |  |
|  |  |  |  |  |  |
|  |  |  |  |  |  |
| **LEVEL B** | | | | | |
| Isometric Press pallof   - Initial position: Standing up with arms outstretched and holding a band which will be extended and fastened on the spalier to one side of the subject. - Exercise:   First movement. Maintaining the distance with the spalier carry out flexion of arms in addition to shoulders extension.  Second movement. Carry out extension of arms in addition to shoulders flexion. | | | | | |
|  |  |  |  |  |  |
|  |  |  |  |  |  |
|  |  |  |  |  |  |
| **LEVEL C** | | | | | |
| Isometric press pallof with displacement.   - Initial position: Standing up with arms outstretched and holding a band which will be extended and fastened on the spalier to one side of the subject. - Exercise:   *First movement.* Maintaining the distance with the spalier carry out flexion of arms in addition to shoulders extension.  *Second movement.* Carry out extension of arms in addition to shoulders flexion. At the same time the subject will separate from spalier doing a side step.  *Third movement.* Back to the initial position doing another side step. | | | | | |
|  |  |  |  |  |  |
|  |  |  |  |  |  |
|  |  |  |  |  |  |
|  |  |  |  |  |  |
|  |  |  |  |  |  |
|  |  |  |  |  |  |

| **Core and stabilisers exercises** | | | | | |
| --- | --- | --- | --- | --- | --- |
| **8. Unilateral Deadlift** | | | | | |
|  | | | | | |
| **LEVEL A** |  | **LEVEL B** |  | **LEVEL C** |  |
| 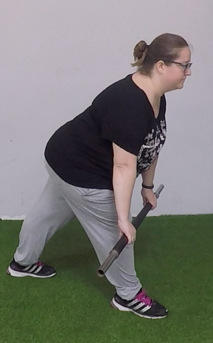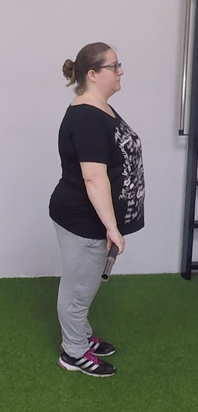 |  | **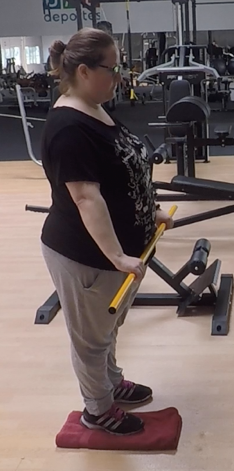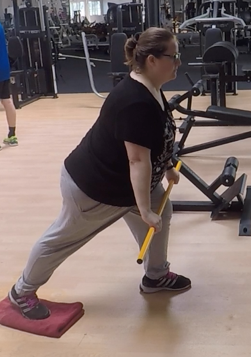** |  | 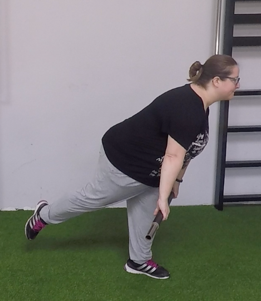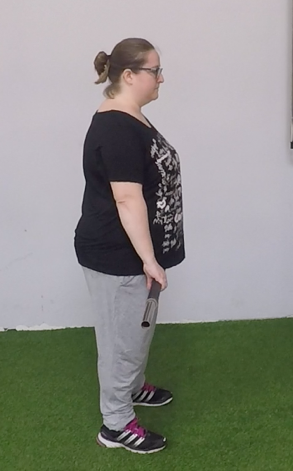 |  |
|  |  |  |  |  |  |
|  |  |  |  |  |  |
|  |  |  |  |  |  |
|  |  |  |  |  |  |
|  |  |  |  |  |  |
|  |  |  |  |  |  |
|  |  |  |  |  |  |
|  |  |  |  |  |  |
|  |  |  |  |  |  |
| **Exercise Descriptions:** | | | | | |
| **LEVEL A** | | | | | |
| Unilateral deadlift without lifting back leg   - Initial position: Stand, erect with feet at shoulder width and hip in neutral position and back straight. With the pike placed just in front of the legs, we reliably grasped a mixed grip (one hand in supination and the other in pronation) with an upper width to the position of the feet. - Exercise:   *Eccentric phase.*  Controlling the descent movement of the pike until arriving with the pike a little below the knee, with the back straight and the hip in a neutral position. Perform a hip and knee flexion with one leg, at the same time, the other leg will perform an extension and will slide back.  *Concentric phase.* Keeping the back straight, we extend the leg, with the other the leg returning to the initial position, until the pike reaches the knees and from there we accompany it with a hip extension until reaching the vertical position of the trunk. | | | | | |
|  |  |  |  |  |  |
|  |  |  |  |  |  |
|  |  |  |  |  |  |
| **LEVEL B** | | | | | |
| Unilateral deadlift with a slider.   - Initial position: Stand, erect with feet at shoulder width (a slider placed in a foot) and hip in neutral position and back straight. With the pike placed just in front of the legs, we reliably grasped a mixed grip (one hand in supination and the other in pronation) with an upper width to the position of the feet. - Exercise:   *Eccentric phase.* Controlling the descent movement of the pike until arriving with the pica a little below the knee, with the back straight and the hip in a neutral position. Perform a hip and knee flexion with one leg at the same time, the other leg which will be support on the slider perform an extension outstretched without separating from the slider.  *Concentric phase.* Keeping the back straight, we extend the leg (with the other the leg still supported on the slider, sliding to return to starting position) until the pike reaches the knees and from there we accompany it with a hip extension until reaching the vertical position. | | | | | |
|  |  |  |  |  |  |
|  |  |  |  |  |  |
|  |  |  |  |  |  |
| **LEVEL C** | | | | | |
| Unilateral deadlift   - Initial position: Stand, erect with feet at shoulder width and hip in neutral position and back straight. With the pike placed just in front of the legs, we reliably grasped a mixed grip (one hand in supination and the other in pronation) with an upper width to the position of the feet. - Exercise:   *Eccentric phase.* Controlling the descent movement of the pike until arriving with the pike a little below the knee, with the back straight and the hip in a neutral position. Perform a hip and knee flexion with one leg at the same time, the other leg will perform an extension and will be suspended in the air parallel with the vertical of the floor.  *Concentric phase.* Keeping the back straight, we extend the leg, with the other the leg returning to touch the ground in the initial position, until the pike reaches the knees and from there we accompany it with a hip extension until reaching the vertical position.  Variant: With a dumbbell in the hand on the side that raises the leg. | | | | | |
|  |  |  |  |  |  |
|  |  |  |  |  |  |
|  |  |  |  |  |  |
|  |  |  |  |  |  |
|  |  |  |  |  |  |
|  |  |  |  |  |  |

| **Core and stabilisers exercises** | | | | | |
| --- | --- | --- | --- | --- | --- |
| **9. Plyometric chest throw** | | | | | |
|  | | | | | |
| **LEVEL A** |  | **LEVEL B** |  | **LEVEL C** |  |
| 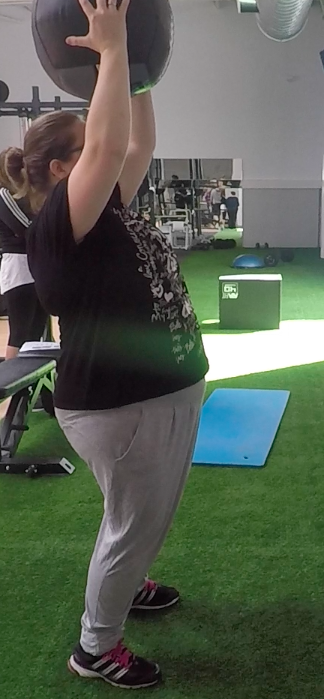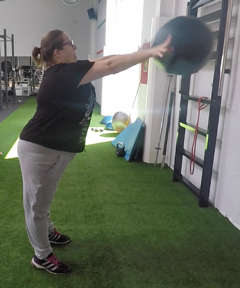 |  | 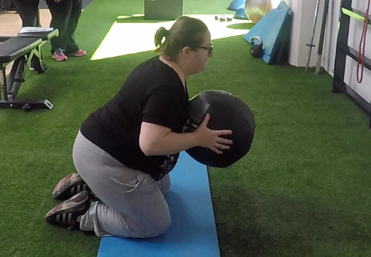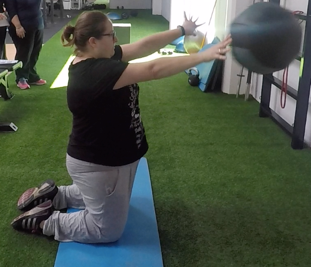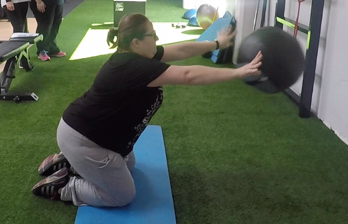 |  | 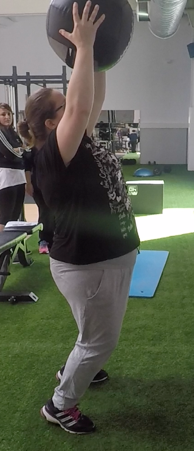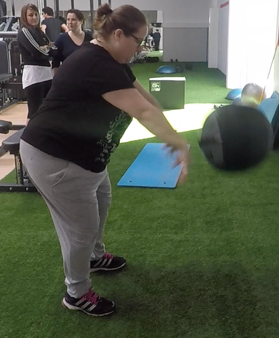 |  |
|  |  |  |  |  |  |
|  |  |  |  |  |  |
|  |  |  |  |  |  |
|  |  |  |  |  |  |
|  |  |  |  |  |  |
|  |  |  |  |  |  |
|  |  |  |  |  |  |
|  |  |  |  |  |  |
|  |  |  |  |  |  |
| **Exercise Descriptions:** | | | | | |
| **LEVEL A** | | | | | |
| Plyometric chest throw.   - Initial position: Standing in front of the wall with the medicine ball at the chest height. - Exercise:   *Concentric phase.* Throw the medicine ball against the wall at the maximum possible speed performing an elbow extension. | | | | | |
|  |  |  |  |  |  |
|  |  |  |  |  |  |
|  |  |  |  |  |  |
| **LEVEL B** | | | | | |
| Plyometric chest throw on knees.   - Initial Position: Down on the knees in front of the wall with the medicine ball at the chest height. - Exercise:   *Concentric phase.* Throw the medicine ball against the wall at the maximum possible speed performing an elbow extension. | | | | | |
|  |  |  |  |  |  |
|  |  |  |  |  |  |
|  |  |  |  |  |  |
| **LEVEL C** | | | | | |
| Plyometric throw to the ground.   - Initial Position: Standing in front of the wall with the medicine ball above the head with arms stretched and with a slight hyper hip extension. - Exercise:   *Concentric phase.* Throw the medicine ball against the floor at the maximum possible speed performing a trunk flexion and shoulder flexo-extension at the same time. | | | | | |
|  |  |  |  |  |  |
|  |  |  |  |  |  |
|  |  |  |  |  |  |

| **Weight-bearing and strength training with elastic bands** | |
| --- | --- |
| **1. Squat with pike** | |
|  | |
| **LEVEL A** |  |
| 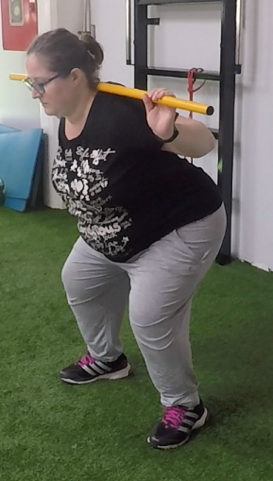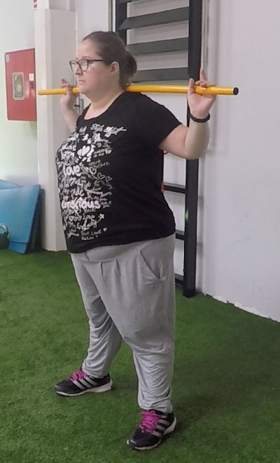 |  |
|  |  |
|  |  |
|  |  |
|  |  |
|  |  |
|  |  |
|  |  |
|  |  |
|  |  |
| **Exercise Descriptions:** | |
| **LEVEL A** | |
| Squat with pike.   - Initial Position: Stand with your feet at shoulder width, or slightly wider and the toes pointed slightly outward. The pike is placed on the trapezoid avoiding that it touches the vertebra C7. - Exercise:   *Eccentric phase.* With the back erect and the hip in a neutral position, a lowering of the body is performed a triple flexion: hip, knee and ankle. The movement should be performed by moving the hips backwards, thus preventing the knees from advancing too far ahead of the tip of the feet  *Concentric phase.* Returning to the starting position in the same way as in the eccentric phase. | |
|  |  |
|  |  |
|  |  |
|  |  |
|  |  |
|  |  |
|  |  |
|  |  |
|  |  |

| **Weight-bearing and strength training with elastic bands** | |
| --- | --- |
| **2. Seated lat Pulldown with band** | |
|  | |
| **LEVEL A** |  |
| 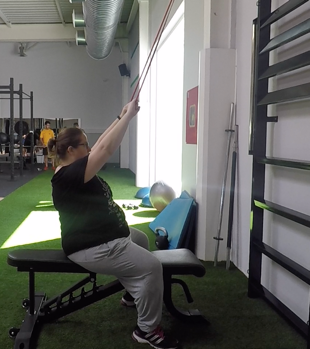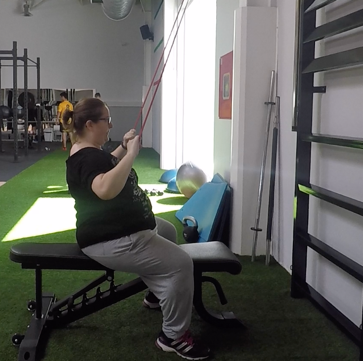 |  |
|  |  |
|  |  |
|  |  |
|  |  |
|  |  |
|  |  |
|  |  |
|  |  |
|  |  |
| **Exercise Descriptions:** | |
| **LEVEL A** | |
| Seated lat Pulldown with band.   1. Initial Position: Sitting with feet flat on the floor, hip in neutral and back straight. We perform a prone band grip greater than the width of the shoulders, with elbows extended and performing shoulder flexion. 2. Exercise:   *Eccentric phase.* Pull the band flexing the elbows and adducting the shoulders until it reaches over the chest.  *Concentric phase.* Controlling the movement of ascent of the band, following the same path we return to the initial position | |
|  |  |
|  |  |
|  |  |
| **LEVEL B** | |
| Seated Lat Pulldown:   1. Initial position: Sitting with feet flat on the floor, hip in neutral and back straight. We perform a prone bar grip greater than the width of the shoulders, with elbows extended and performing shoulder flexion. 2. Exercise:   *Concentric phase.* Pull the band flexing the elbows and adducting the shoulders until it reaches over the chest.  *Eccentric phase.* Controlling the movement of ascent of the bar, following the same path we return to the initial position | |
|  |  |
|  |  |
|  |  |

| **Weight-bearing and strength training with elastic bands** | |
| --- | --- |
| **3. Bench Press with band** | |
|  | |
| **LEVEL A** |  |
| 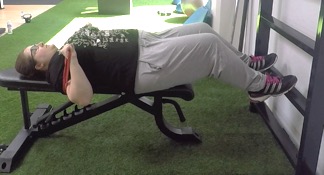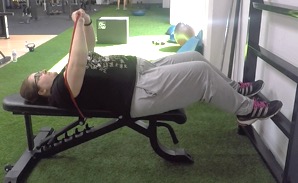 |  |
|  |  |
|  |  |
|  |  |
|  |  |
|  |  |
|  |  |
|  |  |
|  |  |
|  |  |
| **Exercise Descriptions:** | |
| **LEVEL A** | |
| Bench Press with band    - Initial position: In supine position with the hip, back and head in contact with the bench, keeping both feet resting on the floor. We react a prone grip of the band (which is held below the bench) slightly superior to the width of the shoulders, shoulder flexion near 90 º and elbow extension, without reaching the full extension (0 º). - Exercise:   *Eccentric phase.* Perform a lowering of the hands in a controlled way until leaving it a few centimetres from the lower part of the chest, remaining in humeral abduction of 45º and elbow flexion of 90º.  *Concentric phase.* Push the band to the starting position keeping the points of contact with the bench and the floor. | |
|  |  |
|  |  |
|  |  |
|  |  |
|  |  |
|  |  |
|  |  |
|  |  |
|  |  |

| **Weight-bearing and strength training with elastic bands** | |
| --- | --- |
| **4. Pull T-band** | |
|  | |
| **LEVEL A** |  |
| 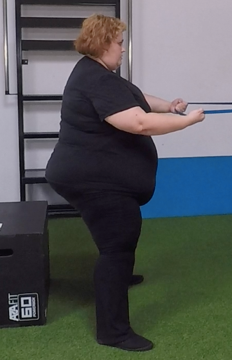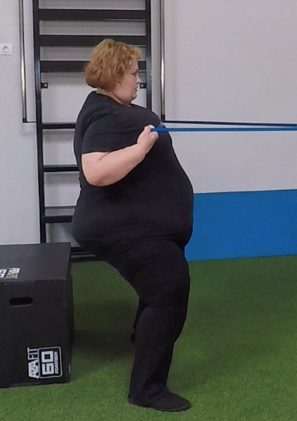 |  |
|  |  |
|  |  |
|  |  |
|  |  |
|  |  |
|  |  |
|  |  |
|  |  |
|  |  |
| **Exercise Descriptions:** | |
| **LEVEL A** | |
| Pull T-band   1. Initial position: Standing with knees slightly bent and arms stretched forward at shoulder height by gripping an elastic band 2. Exercise:   *Concentric phase.* perform scapular retraction in addition to a shoulder extension with an elbow flexion.  *Eccentric phase.* Back to the initial position following the path of the concentric phase. | |
|  |  |
|  |  |
|  |  |

| **Weight-bearing and strength training with elastic bands** | |
| --- | --- |
| **5. Push press with pike** | |
|  | |
| **LEVEL A** |  |
| 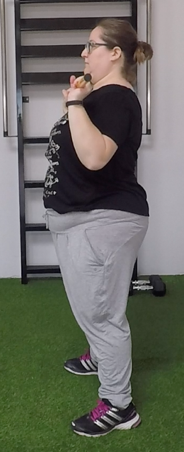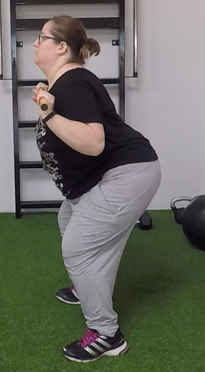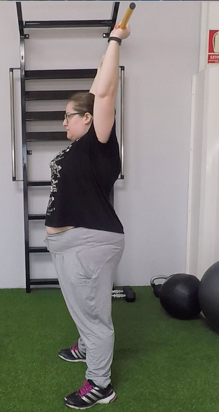 |  |
|  |  |
|  |  |
|  |  |
|  |  |
|  |  |
|  |  |
|  |  |
|  |  |
|  |  |
| **Exercise Descriptions:** | |
| **LEVEL A** | |
| Push press with pike   1. Initial position: Standing with your feet at shoulder height, place the pike in front of your shoulders, holding it with your hands in a prone position at shoulder width, with your back erect. 2. Exercise:   *Eccentric phase from the starting position*: Perform a small leg and hip flexion, keeping your back erect and the pike in the starting position.  *Eccentric phase.* Without having made any stops between the previous phase and the concentric, carry out an upward push with the legs (extension of hip, knee and ankle), while raising the pike above the head until the complete extension of the elbow and maintaining the bar lined with the hips.  *Concentric phase.* Lowering the pike to the initial position of the bar following the same path up to shoulder height, accompanying the movement with a small leg and hip flexion. | |
|  |  |
|  |  |
|  |  |

| **Weight-bearing and strength training with elastic bands** | |
| --- | --- |
| **6. Deadlift with pike** | |
|  | |
| **LEVEL A** |  |
| 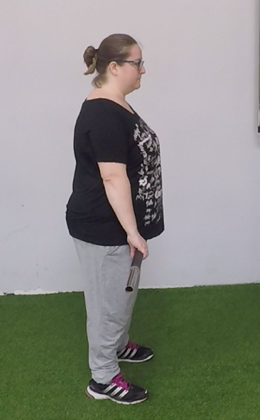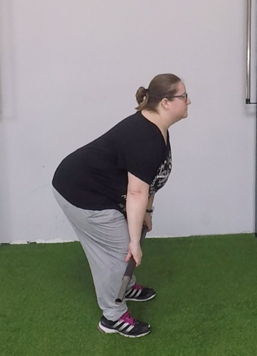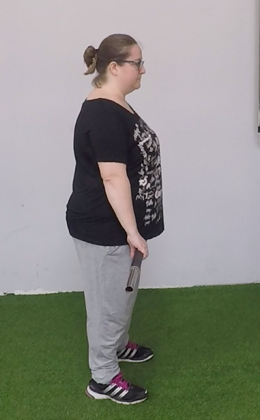 |  |
| **Exercise Descriptions:** | |
| **LEVEL A** | |
| Deadlift with pike.   - Initial position: Stand, erect with feet at shoulder width and hip in neutral position and back straight. With the pike placed just in front of the legs, we reliably grasped a mixed grip (one hand in supination and the other in pronation) with an upper width to the position of the feet. - Exercise:   *Eccentric phase.* Controlling the descent movement of the pike, with the back straight and the hip in a neutral position. Perform a hip and knee flexion at the same time, until arriving with the pike a little below the knee; we will stop sooner if lumbar lordosis disappears.  *Concentric phase.* Keeping the back straight, we extend the legs until the pike reaches the knees and from there we accompany it with a hip extension until reaching the vertical position of the trunk. | |
|  |  |
|  |  |
|  |  |

| Strength training exercises | | | | | | |  |
| --- | --- | --- | --- | --- | --- | --- | --- |
|  | | | | | | |  |
| **1. Squat** |  | **2. Seated Lat Pull-down** |  | **3. Bench Press** |  | **4. Seated Low Row (LF Cable)** |  |
| 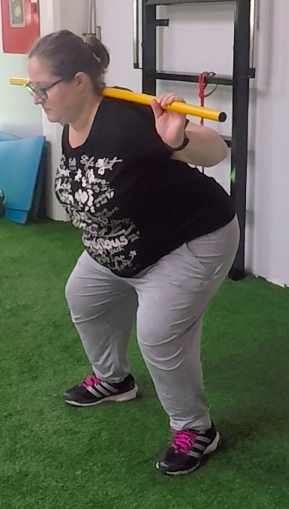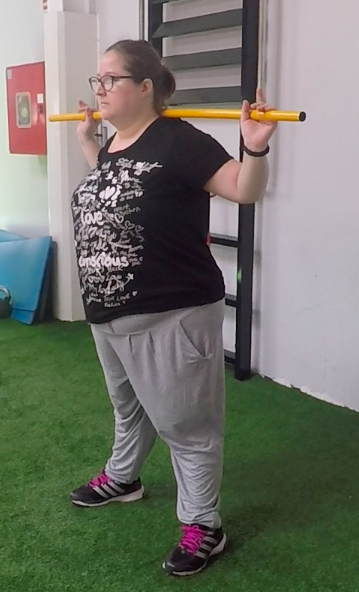 |  | 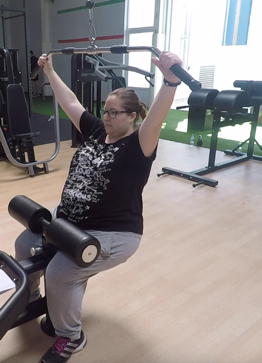  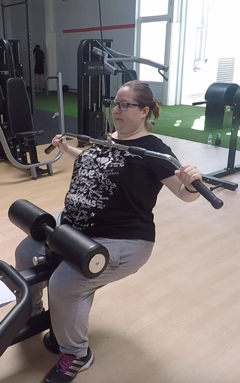 |  | 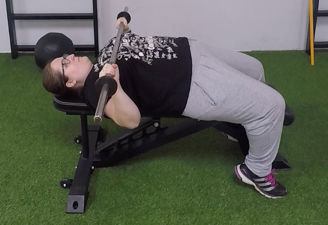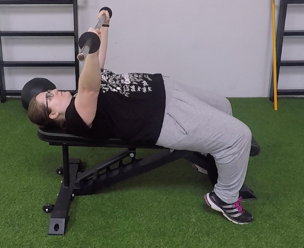 |  | 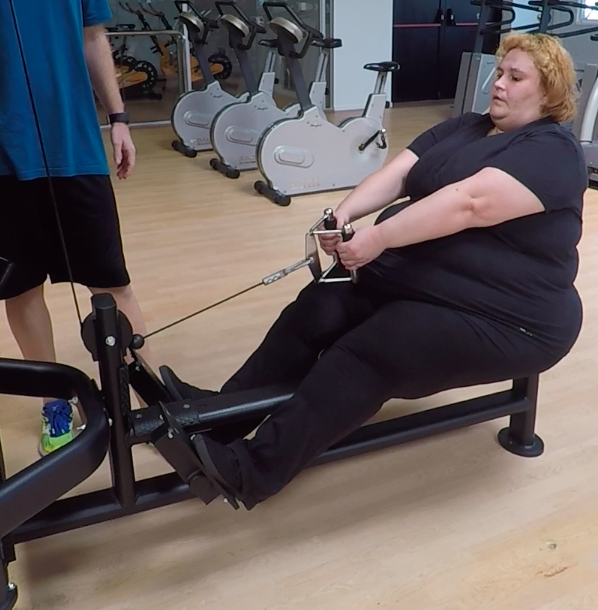 |  |
| **5.**  **Push press with dumbbells.** |  | **6. Deadlift** |  |  |  |  |  |
|  |  |  |  |  |  |  |  |
| Exercises Description | | | | | | |  |
| **1. Squat** | | | | | | |  |
| 1. Muscles involved: Quadriceps, glutes and hamstrings. 2. Initial position: Stand with your feet at shoulder width, or slightly wider and the toes pointed slightly outward. The bar is placed on the trapezoid avoiding that it touches the vertebra C7. 3. Exercise performance:  *Eccentric phase.* With the back erect and the hip in a neutral position, a lowering of the body is performed by a triple flexion: hip, knee and ankle. The movement should be performed by moving the hips backwards, thus preventing the knees from advancing too far ahead of the knees.   *Concentric phase.* We return to the starting position in the same way as in the eccentric phase. | | | | | | |  |
| **2. Seated Lat Pull-down** | | | | | | |  |
| 1. Muscle involved: latissimus dorsi, biceps brachial, larger round. 2. Initial position: Sitting with feet flat on the floor, hip in neutral and back straight. We perform a prone bar grip greater than the width of the shoulders, with elbows extended and performing shoulder flexion. 3. Exercise performance:  *Concentric phase.* Pull the bar by flexing and adducting the arm until it reaches over the chest.   *Eccentric phase.* Controlling the movement of ascent of the bar, following the same path we return to the initial position. | | | | | | |  |
| **3. Bench Press or Adapted Push Ups** | | | | | | |  |
| - Muscles involved: Pectoral, anterior deltoid, triceps, brachial, serrated. - Initial position: In supine position with the hip, back and head in contact with the bench, keeping both feet resting on the floor. We react a prone grip of the bar, slightly superior to the width of the shoulders, shoulder flexion near 90 º and elbow extension, without reaching the full extension (0º) - Exercise performance:  *Eccentric phase.* Perform a lowering of the bar in a controlled way until leaving it a few centimetres from the lower part of the chest, remaining in humeral abduction of 45º and elbow flexion of 90º.   *Concentric phase.* Push the bar to the starting position keeping the points of contact with the bench and the floor.  * If some patients cannot do the bench press by shoulders problems, the alternative exercise will be push ups in the wall with inclination progression.   - Muscle involved: Pectoral, anterior deltoid, triceps, brachial, serrated. - Initial Position: Stand separated from the wall, tilted forward with arms outstretched and hands resting on the wall, placed a little more open than the shoulders width. - Exercise performance:   *Eccentric phase.* Perform a lowering of the body, performing humeral abduction and elbow flexion in a controlled way until leaving the face a few centimetres from the wall.  *Concentric phase.* Push the body to the starting position. | | | | | | |  |
| **4. Seated Low Row (LF Cable)** | | | | | | |  |
| 1. Muscles involved: latissimus dorsal, biceps brachial, larger round, deltoid posterior, trapezoid medium portion. 2. Initial position: Sitting with feet flat on the floor, hip in neutral and back straight. We perform a prone bar grip greater than the width of the shoulders, with elbows extended and performing shoulder flexion. 3. Exercise performance:  *Concentric phase.* Pull the bar by flexing and adducting the arm until it reaches over the chest.   *Eccentric phase.* Controlling the movement of ascent of the bar, following the same path we return to the initial position. | | | | | | |  |
| **5. Push Press with dumbbells** | | | | | | |  |
| 1. Muscles involved: Gluteus, hamstrings, quadriceps, gastrocnemius, soleus, deltoids. 2. Initial position: Standing with your feet at shoulder height, place the dumbbells in front of your shoulders, holding it with your hands in a supine position at shoulder width, with your back erect. 3. Exercise performance:  *Eccentric phase from the starting position:* Perform a small leg and hip flexion, keeping your back erect and the bar in the starting position.   *Concentric phase.* Without having made any stops between the previous phase and the concentric, carry out an upward push with the legs (extension of hip, knee and ankle), while raising the dumbbells above the head until the complete extension of the elbow, at the same time that stretched the arms a prone grip change will be made and maintaining the dumbbells lined with hips.  *Eccentric phase.* Lowering the pike to the initial position of the bar following the same path up to shoulder height, accompanying the movement with a small leg and hip flexion. | | | | | | |  |
| **6. Deadlift** | | | | | | |  |
| 1. Muscles involved: Quadriceps, glutes, hamstrings and lumbar muscles. 2. Initial position: Feet shoulder width with knees bent close to 90 °, hip flexion with trunk in neutral position and back straight. With the bar placed just in front of the legs, we reliably grasped a mixed grip (one hand in supination and the other in pronation) with an upper width to the position of the feet. 3. Exercise performance:  *Concentric phase.* Keeping the back straight, we extend the legs until the bar reaches the knees and from there we accompany it with a hip extension until reaching the vertical position   *Eccentric phase.* Controlling the descent movement of the bar, following the same path we return to the starting position. | | | | | | |  |
| Aerobic training exercises | | | | | | |  |
|  | | | | | | |  |
| **Treadmill** | | | | | | | |
|  | | | | | | | |
| Exercise Description | | | | | | |  |
| **Treadmill** | | | | | | |  |
| Walk or run with the back erect and looking forward, on treadmill to the intensity needed for each person. Trying to maintain a neutral hip position and not performing knee valgus. | | | | | | |  |

| Static stretching exercises | | | | | | |
| --- | --- | --- | --- | --- | --- | --- |
|  | | | | | | |
| 1 |  | 2 |  | 3 |  | 4 |
|  |  |  |  |  |  |  |
| 5 |  | 6 |  | 7 |  | 8 |
|  |  | Recommended from the eighth week |  |  |  |  |
|  |  |  |  |  |  |  |
|  |  |  |  |  |  |  |
| Exercises Description | | | | | | |
|  | | | | | | |
| 1. Perform an adduction and flexion of shoulder grabbing the arm with the opposite hand, until reaching the maximum range of movement, without it hurting. | | | | | | |
|  | | | | | | |
| 2. Perform an abduction shoulder with elbow flexed grabbing the arm with the opposite hand, until reaching the maximum range of movement, without it hurting. | | | | | | |
|  | | | | | | |
| 3. Make a flexion of a shoulder, raising the arm and flexing the elbow. Stretch the triceps helping with the support of the other hand. | | | | | | |
|  | | | | | | |
| 4. Clinging to a spailer perform a trunk flexion. Pushing with the body back until reaching the maximum range of movement, without it hurting. | | | | | | |
|  | | | | | | |
| 5. Perform a hip and trunk flexion, until reaching the maximum range of movement, without it hurting. | | | | | | |
|  | | | | | | |
| 6. Perform a knee flexion, with hip in retroversion, grabbing the leg with the hand until reaching the maximum range of movement, without it hurting. | | | | | | |
|  | | | | | | |
| 7. Clinging to a spailer perform with a leg outstretched an abduction and with the other a knee flexion carry the stretched leg down as far as possible, without it hurting. | | | | | | |
|  | | | | | | |
| 8. Perform a hip flexion with one leg outstretched and the other flexed, with trunk in a neutral position, until reaching the maximum range of movement, without it hurting. | | | | | | |
