# Supplementary material for: Supervised exercise following bariatric surgery in morbid obese adults: CERT-based exercise study protocol of the EFIBAR randomised controlled trial
Source: BMC Surg. 2019 Sep 5;19:127. doi: 10.1186/s12893-019-0566-9 (PMC6729089; doi:10.1186/s12893-019-0566-9)
Supplement: Supplementary file 3 — Table S3. Detailed tally sheet used to assess adherence to exercise programme (Item 5), type and number of adverse events that occur during exercise (Item 11), and fidelity (Item 16a and 16b). (DOCX 29 kb) [file 12893_2019_566_MOESM3_ESM.docx]

| **Additional file 3: Table S3.** Detailed tally sheet used to assess adherence to exercise programme (Item 5), type and number of adverse events that occur during exercise (Item 11), and fidelity (Item 16a and 16b). |
| --- |

| **ID:** |  | **Session number:** |  | **Time:** | |  | | | | | | |  | | | **Date:** | | | | |  | |  |  | | |  |  | |  | |
| --- | --- | --- | --- | --- | --- | --- | --- | --- | --- | --- | --- | --- | --- | --- | --- | --- | --- | --- | --- | --- | --- | --- | --- | --- | --- | --- | --- | --- | --- | --- | --- |
| ***Attendance*** | **Yes** | **No** |  |  | |  | |  | |  | | |  | | |  | | | | |  | |  |  | | |  |  | |  | |
| Attend the training session |  |  |  | | | | | | | | | | | | | | | | | | | | | | | | | | | | |
| Attend on time |  |  |  | | | | | | | | | | | | | | | | | | | | | | | | | | | | |
| ***Mood feeling before this training session*** | **Feeling Scale** | |  |  | | |  |  | |  | | |  | | | |  | | | |  | |  |  | | |  |  | |  | |
|  |  |  |  | **-5** | | | **-4** | **-3** | | **-2** | | | **-1** | | | | **0** | | | | **1** | | **2** | **3** | | | **4** | **5** | |  | |
| ***Physically drained feeling before this training session*** | **HPHEE Scale** | |  | **Very Bad** | | |  | **Bad** | |  | | | **Fairly Bad** | | | | **Neutral** | | | | **Fairly Good** | |  | **Good** | | |  | **Very Good** | |  | |
|  |  |  |  |  | | |  |  | |  | | |  | | | |  | | | |  | |  |  | | |  |  | |  | |
|  |  |  |  | **0** | | | **1** | | **2** | | | **3** | | | **4** | | | | **5** | **6** | | **7** | | **8** | | **9** | | | **10** | |  |
| ***Extra physical activity*** | **Yes** | **No** |  | **Nothing** | | |  |  | |  | | |  | | |  | | | | |  | |  |  | | |  | | **Totally** | |  |
|  |  |  |  |  | | |  |  | |  | | |  | | |  | | | | |  | |  |  | | |  | |  | |  |
|  | **Frequency** | **Minutes** | **Type** | | | | |  | |  | | |  | | |  | | | | |  | |  |  | | |  | |  | |  |
|  |  |  |  | | | | |  | |  | | |  | | |  | | | | |  | |  |  | | |  | |  | |  |
| ***Session training parts*** | | | | | | | | | | | | | | | | | | | | | | | | | | | | | | | |
| ***Warm-up*** | **Yes** | **No** |  |  | |  | |  | |  | | |  | | |  | | | | | | | | | | | | | | | |
| Complete the overall warm-up part (minutes) |  |  |  | | | | | | | | | |  | | |  | | | | |  | |  |  | | |  |  | |  | |
| Compliant attitude during warm-up |  |  |  | **HR (bpm)** | | | | **Time** | | | | |  | | |  | | | | |  | |  |  | | |  |  | |  | |
| RPE (0-10) immediately after warm-up |  |  |  |  | | | |  | | | | |  | | |  | | | | |  | |  |  | | |  |  | |  | |
|  |  |  |  |  | |  | |  | |  | | |  | | |  | | | | |  | |  |  | | |  |  | |  | |
| ***Strength training*** | **Yes** | **No** |  |  | |  | |  | |  | | |  | | |  | | | | | | | | | | | | | | | |
| Complete the overall resistance part (exercises) |  |  |  |  | |  | |  | |  | | |  | | |  | | | | | | | | | | | | | | | |
| Compliant attitude during resistance part |  |  |  | **HR (bpm)** | | | | **Time** | | | | |  | | |  | | | | | | | | | | | | | | | |
| ONMI resistance scale (0-10) immediately after resistance part |  |  |  |  | | | |  | | | | |  | | |  | | | | |  | |  |  | | |  |  | |  | |
|  |  |  |  |  | |  | |  | |  | | |  | | |  | | | | |  | |  |  | | |  |  | |  | |
| ***Aerobic training*** | **Yes** | **No** |  |  | |  | |  | |  | | |  | | |  | | | | |  | |  |  | | |  |  | |  | |
| Complete overall cardiovascular part (minutes) |  |  |  |  | |  | |  | |  | | |  | | |  | | | | |  | |  |  | | |  |  | |  | |
| Compliant attitude during cardiovascular part |  |  |  | **HR (bpm)** | | | | **Time** | | | | |  | | |  | | | | | | | | | | | | | | | |
| RPE (0-10) immediately after cardiovascular part |  |  |  |  | | | |  | | | | |  | | |  | | | | |  | |  |  | | |  |  | |  | |
|  |  |  |  |  | |  | |  | |  | | |  | | |  | | | | |  | |  |  | | |  |  | |  | |
| ***Cool down*** | **Yes** | **No** |  |  | |  | |  | |  | | |  | | |  | | | | |  | |  |  | | |  |  | |  | |
| Complete overall calm down part (exercises) |  |  |  |  | |  | |  | |  | | |  | | |  | | | | |  | |  |  | | |  |  | |  | |
| Compliant attitude during calm down part |  |  |  | **HR (bpm)** | | | | **Time** | | | | |  | | |  | | | | | | | | | | | | | | | |
|  |  |  |  |  | |  | |  | | | | |  | | |  | | | | |  | |  |  | | |  |  | |  | |
| **RPE Session** |  |  |  |  | | | |  | | | | |  | | |  | | | | |  | |  |  | | |  |  | |  | |
|  |  |  |  |  | |  | |  | |  | | |  | | |  | | | | | | | | | | | | | | | |
| ***Overall training session*** | **Yes** | **No** |  |  | |  | |  | |  | | |  | | |  | | | | |  | |  |  | | |  |  | |  | |
| Pulsometer working well in overall training session |  |  |  |  | |  | |  | |  | | |  | | |  | | | | |  | |  |  | | |  |  | |  | |
| Complete the overall session |  |  |  |  | |  | |  | |  | | |  | | |  | | | | |  | |  |  | | |  |  | |  | |
| Number of adverse events that occur |  |  |  |  | |  | |  | |  | | |  | | |  | | | | |  | |  |  | | |  |  | |  | |
| Type of adverse events that occur |  | | | | | | | | | | | | | | | | | | | | | | | | | | | | | | |
|  |  |  |  |  | |  | |  | |  | | |  | | |  | | | | | | | | | | | | | | | |
| ***Mood feeling after this training session*** | **Feeling Scale** | |  | **-5** | **-4** | | | **-3** | | | **-2** | | | **-1** | | | | **0** | | | **1** | | **2** | **3** | | **4** | | | **5** | |  |
|  |  |  |  | **Very Bad** |  | | | **Bad** | | |  | | | **Fairly Bad** | | | | **Neutral** | | | **Fairly Good** | |  | **Good** | |  | | | **Very Good** | |  |
| ***Observations:*** |  |  |  |  | |  | |  | |  | | |  | | |  | | | | |  | |  |  | | |  |  | |  | |
|  | | | | | | | | | | | | |  | | | **Instructor** | | | | | | |  | | **Yes** | | | | **No** | |  |
|  |  |  |  |  |  |  |  |  |  |  |  |  |  | | | Same instructor last sessions? | | | | | | |  | |  | | | |  | |  |
|  |  |  |  |  |  |  |  |  |  |  |  |  |  | | | Match with another participant? | | | | | | |  | |  | | | |  | |  |

**Table S4.** Detailed tally sheet used to assess adherence to exercise programme (Item 5), type and number of adverse events that occur during exercise (Item 11), and fidelity (Item 16a and 16b).

| **ID:** |  | **Session number:** |  | **Time:** | |  | | | | | | |  | | | **Date:** | | | | |  | |  |  | | |  |  | |  | |
| --- | --- | --- | --- | --- | --- | --- | --- | --- | --- | --- | --- | --- | --- | --- | --- | --- | --- | --- | --- | --- | --- | --- | --- | --- | --- | --- | --- | --- | --- | --- | --- |
| ***Attendance*** | **Yes** | **No** |  |  | |  | |  | |  | | |  | | |  | | | | |  | |  |  | | |  |  | |  | |
| Attend the training session |  |  |  | | | | | | | | | | | | | | | | | | | | | | | | | | | | |
| Attend on time |  |  |  | | | | | | | | | | | | | | | | | | | | | | | | | | | | |
| ***Mood feeling before this training session*** | **Feeling Scale** | |  |  | | |  |  | |  | | |  | | | |  | | | |  | |  |  | | |  |  | |  | |
|  |  |  |  | **-5** | | | **-4** | **-3** | | **-2** | | | **-1** | | | | **0** | | | | **1** | | **2** | **3** | | | **4** | **5** | |  | |
| ***Physically drained feeling before this training session*** | **HPHEE Scale** | |  | **Very Bad** | | |  | **Bad** | |  | | | **Fairly Bad** | | | | **Neutral** | | | | **Fairly Good** | |  | **Good** | | |  | **Very Good** | |  | |
|  |  |  |  |  | | |  |  | |  | | |  | | | |  | | | |  | |  |  | | |  |  | |  | |
|  |  |  |  | **0** | | | **1** | | **2** | | | **3** | | | **4** | | | | **5** | **6** | | **7** | | **8** | | **9** | | | **10** | |  |
| ***Extra physical activity*** | **Yes** | **No** |  | **Nothing** | | |  |  | |  | | |  | | |  | | | | |  | |  |  | | |  | | **Totally** | |  |
|  |  |  |  |  | | |  |  | |  | | |  | | |  | | | | |  | |  |  | | |  | |  | |  |
|  | **Frequency** | **Minutes** | **Type** | | | | |  | |  | | |  | | |  | | | | |  | |  |  | | |  | |  | |  |
|  |  |  |  | | | | |  | |  | | |  | | |  | | | | |  | |  |  | | |  | |  | |  |
| ***Session training parts*** | | | | | | | | | | | | | | | | | | | | | | | | | | | | | | | |
| ***Warm-up*** | **Yes** | **No** |  |  | |  | |  | |  | | |  | | |  | | | | | | | | | | | | | | | |
| Complete the overall warm-up part (minutes) |  |  |  | | | | | | | | | |  | | |  | | | | |  | |  |  | | |  |  | |  | |
| Compliant attitude during warm-up |  |  |  | **HR (bpm)** | | | | **Time** | | | | |  | | |  | | | | |  | |  |  | | |  |  | |  | |
| RPE (0-10) immediately after warm-up |  |  |  |  | | | |  | | | | |  | | |  | | | | |  | |  |  | | |  |  | |  | |
|  |  |  |  |  | |  | |  | |  | | |  | | |  | | | | |  | |  |  | | |  |  | |  | |
| ***Strength training*** | **Yes** | **No** |  |  | |  | |  | |  | | |  | | |  | | | | | | | | | | | | | | | |
| Complete the overall resistance part (exercises) |  |  |  |  | |  | |  | |  | | |  | | |  | | | | | | | | | | | | | | | |
| Compliant attitude during resistance part |  |  |  | **HR (bpm)** | | | | **Time** | | | | |  | | |  | | | | | | | | | | | | | | | |
| ONMI resistance scale (0-10) immediately after resistance part |  |  |  |  | | | |  | | | | |  | | |  | | | | |  | |  |  | | |  |  | |  | |
|  |  |  |  |  | |  | |  | |  | | |  | | |  | | | | |  | |  |  | | |  |  | |  | |
| ***Aerobic training*** | **Yes** | **No** |  |  | |  | |  | |  | | |  | | |  | | | | |  | |  |  | | |  |  | |  | |
| Complete overall cardiovascular part (minutes) |  |  |  |  | |  | |  | |  | | |  | | |  | | | | |  | |  |  | | |  |  | |  | |
| Compliant attitude during cardiovascular part |  |  |  | **HR (bpm)** | | | | **Time** | | | | |  | | |  | | | | | | | | | | | | | | | |
| RPE (0-10) immediately after cardiovascular part |  |  |  |  | | | |  | | | | |  | | |  | | | | |  | |  |  | | |  |  | |  | |
|  |  |  |  |  | |  | |  | |  | | |  | | |  | | | | |  | |  |  | | |  |  | |  | |
| ***Cool down*** | **Yes** | **No** |  |  | |  | |  | |  | | |  | | |  | | | | |  | |  |  | | |  |  | |  | |
| Complete overall calm down part (exercises) |  |  |  |  | |  | |  | |  | | |  | | |  | | | | |  | |  |  | | |  |  | |  | |
| Compliant attitude during calm down part |  |  |  | **HR (bpm)** | | | | **Time** | | | | |  | | |  | | | | | | | | | | | | | | | |
|  |  |  |  |  | |  | |  | | | | |  | | |  | | | | |  | |  |  | | |  |  | |  | |
| **RPE Session** |  |  |  |  | | | |  | | | | |  | | |  | | | | |  | |  |  | | |  |  | |  | |
|  |  |  |  |  | |  | |  | |  | | |  | | |  | | | | | | | | | | | | | | | |
| ***Overall training session*** | **Yes** | **No** |  |  | |  | |  | |  | | |  | | |  | | | | |  | |  |  | | |  |  | |  | |
| Pulsometer working well in overall training session |  |  |  |  | |  | |  | |  | | |  | | |  | | | | |  | |  |  | | |  |  | |  | |
| Complete the overall session |  |  |  |  | |  | |  | |  | | |  | | |  | | | | |  | |  |  | | |  |  | |  | |
| Number of adverse events that occur |  |  |  |  | |  | |  | |  | | |  | | |  | | | | |  | |  |  | | |  |  | |  | |
| Type of adverse events that occur |  | | | | | | | | | | | | | | | | | | | | | | | | | | | | | | |
|  |  |  |  |  | |  | |  | |  | | |  | | |  | | | | | | | | | | | | | | | |
| ***Mood feeling after this training session*** | **Feeling Scale** | |  | **-5** | **-4** | | | **-3** | | | **-2** | | | **-1** | | | | **0** | | | **1** | | **2** | **3** | | **4** | | | **5** | |  |
|  |  |  |  | **Very Bad** |  | | | **Bad** | | |  | | | **Fairly Bad** | | | | **Neutral** | | | **Fairly Good** | |  | **Good** | |  | | | **Very Good** | |  |
| ***Observations:*** |  |  |  |  | |  | |  | |  | | |  | | |  | | | | |  | |  |  | | |  |  | |  | |
|  | | | | | | | | | | | | |  | | | **Instructor** | | | | | | |  | | **Yes** | | | | **No** | |  |
|  |  |  |  |  |  |  |  |  |  |  |  |  |  | | | Same instructor last sessions? | | | | | | |  | |  | | | |  | |  |
|  |  |  |  |  |  |  |  |  |  |  |  |  |  | | | Match with another participant? | | | | | | |  | |  | | | |  | |  |
